# Photodynamic Therapy: Past, Current, and Future

**DOI:** 10.3390/ijms252011325

**Published:** 2024-10-21

**Authors:** David Aebisher, Sara Czech, Klaudia Dynarowicz, Maciej Misiołek, Katarzyna Komosińska-Vassev, Aleksandra Kawczyk-Krupka, Dorota Bartusik-Aebisher

**Affiliations:** 1 Department of Photomedicine and Physical Chemistry, Medical College, The Rzeszów University, 35-959 Rzeszów, Poland; 2 Department of Biochemistry and General Chemistry, Medical College, The Rzeszów University, 35-959 Rzeszów, Poland; sc126240@stud.ur.edu.pl (S.C.); dbartusikaebisher@ur.edu.pl (D.B.-A.); 3 Center for Innovative Research in Medical and Natural Sciences, Medical College, The Rzeszów University, 35-959 Rzeszów, Poland; kdynarowicz@ur.edu.pl; 4 Department of Otorhinolaryngology and Oncological Laryngology in Zabrze, Medical University of Silesia, 40-055 Katowice, Poland; maciej.misiolek@sum.edu.pl; 5 Department of Clinical Chemistry and Laboratory Diagnostics, Faculty of Pharmaceutical Sciences in Sosnowiec, Medical University of Silesia in Katowice, 40-055 Katowice, Poland; kvassev@sum.edu.pl; 6 Department of Internal Medicine, Angiology and Physical Medicine, Center for Laser Diagnostics and Therapy, Medical University of Silesia in Katowice, 40-055 Katowice, Poland; akawczyk@gmail.com

**Keywords:** PDT, photomedicine, phototherapy

## Abstract

The Greek roots of the word “photodynamic” are as follows: “phos” (φ$\overset{\sim }{\omega }$ς) means “light” and “dynamis” (δύναμις) means “force” or “power”. Photodynamic therapy (PDT) is an innovative treatment method based on the ability of photosensitizers to produce reactive oxygen species after the exposure to light that corresponds to an absorbance wavelength of the photosensitizer, either in the visible or near-infrared range. This process results in damage to pathological cancer cells, while minimizing the impact on healthy tissues. PDT is a promising direction in the treatment of many diseases, with particular emphasis on the fight against cancer and other diseases associated with excessive cell growth. The power of light contributed to the creation of phototherapy, whose history dates back to ancient times. It was then noticed that some substances exposed to the sun have a negative effect on the body, while others have a therapeutic effect. This work provides a detailed review of photodynamic therapy, from its origins to the present day. It is surprising how a seemingly simple beam of light can have such a powerful healing effect, which is used not only in dermatology, but also in oncology, surgery, microbiology, virology, and even dentistry. However, despite promising results, photodynamic therapy still faces many challenges. Moreover, photodynamic therapy requires further research and improvement.

## 1. Introduction

**Figure 1:**
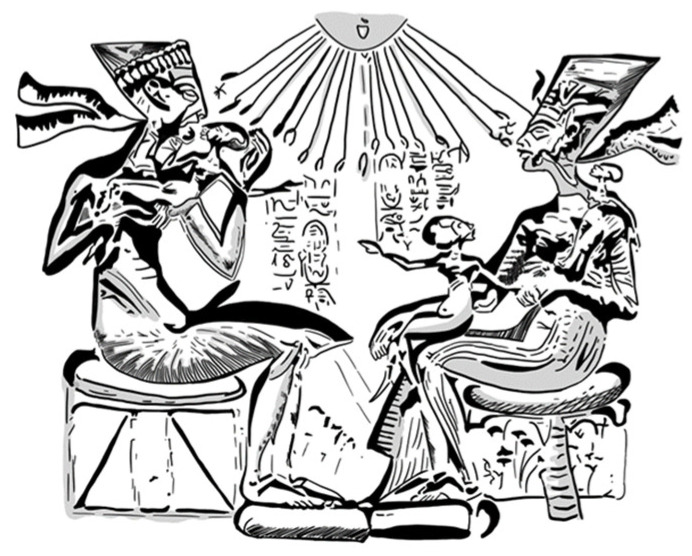
Heliotherapy in ancient times. This figure is a historical image reproduction carried out by author (Sara Czech). It is based on Ref. [5].

Sunlight healing, known as heliotherapy, was practiced by many ancient cultures around the world [1,2]. For example, the Greeks preferred a form of heliotherapy in which participants lay naked in specially designated areas, subjected to total body exposure to the sun, called arenation. The Greek physician Herodotus is considered one of the pioneers of this method (Figure 1). His teaching emphasized the usefulness of sun exposure for restoring health. The Egyptians, Chinese, and Indians also believed in the beneficial effects of sunlight on overall health and attempted to cure various diseases using this natural light source, including rickets, psoriasis, and psychoses. In addition, light helped the Egyptians mask symptoms of vitiligo—they used juice from the Egyptian amine fruit, which, after exposure to solar radiation, imitated a natural tan [3,4]. Over time, together with the advent of Christianity, both sun worship and the use of sunlight as a method of healing came to be considered pagan practices, although they continued in various forms.

Important events in the history of phototherapy were the discovery and description of infrared radiation by Herschel in 1800 and ultraviolet radiation by Ritter in 1806 [6]. At the turn of the 18th and 19th centuries, light began to be used in France as a form of therapy to treat various diseases. At that time, light was used to treat diseases such as tuberculosis, psoriasis, scurvy, rheumatism, paralysis, and muscle weakness. In the 19th century, the bactericidal properties of ultraviolet radiation were discovered [7]. This discovery was groundbreaking in treating infections, and ultraviolet light therapy became popular. The Danish researcher Niels Finsen used this knowledge and used an arc lamp to irradiate patients suffering from cutaneous tuberculosis (lapus vulgaris). His research work and the use of light therapy brought excellent results, for which he was awarded the Nobel Prize in medicine in 1903 [8]. Finsen, born in the Faroe Islands, was also interested in studying the influence of light on living organisms. He performed experiments on light and published a paper in 1893 on the treatment of smallpox [9]. Oscar Raab, who worked in the laboratory of Professor Herman von Tappeiner in Munich, performed research on the use of acridine dyes, Raab noticed that the results of an experiment performed on paramecium colonies varied depending on conditions such as the time of day and the amount of light (in 1902, Ledoux-Lebards discovered that oxygen was also necessary in order to achieve this effect). After many attempts, he described the basics of the theory of photodynamic reactions [10,11]. In the following years, Oscar Raab continued his research on the application of photodynamic reactions in collaboration with his teacher, Hermann von Tappeiner. Their research focused, among others, on the use of this method in the treatment of skin lesions associated with tuberculosis and syphilis. In 1903, Tappeiner and a dermatologist named Jesionek used a combination of eosin and light application in the treatment of skin cancer [12]. We also owe Tappeiner for the introduction of the term “photodynamic” therapy, i.e., light-dependent therapy. In 1913, the German researcher Friedrich Meyer-Betz decided to investigate whether a similar phenomenon also occurred in humans. He gave himself 200 mg of hematoporphyrin intravenously and then exposed his skin to sunlight. The effect of this experiment was increased swelling, pain, and itching in the areas that were irradiated [11,12]. One of the most important reports was the selectivity of hematoporphyrin towards cancer cells, described by Palikard in 1924. Subsequent years of experience led to the creation of a derivative of hematoporphyrin, HpD (English: hematoporphyrin derivative), which is a mixture of monomers, dimers, and higher oligomers joined by ester and ether linkages with an even greater selectivity towards cancer cells. In the years 1942–1948, scientists Auler, Banzer, Figge, and Weiland conducted research on the use of porphyrins in diagnosis and treatment using the photodynamic method. In 1942, Auler and Banzer studied porphyrins administered to the body, which led to the further understanding of their potential use in PDT. In 1948, Figge and Weiland continued research on various porphyrin derivatives and searched for their applications in diagnostics and therapy using the photodynamic method. In 1955, Rasmussen-Taxdal and colleagues described the usefulness of a diagnostic method that involved the intravenous administration of a photosensitizer (PS), enabling tumor localization during the procedure operational. It was not until 1972 that studies conducted in a mouse model of glioma were published, in which tumor growth was inhibited even up to twenty days after the use of PDT [13]. In 1975, Kelly and his colleagues performed effective PDT in a patient with bladder cancer [14]. At the same time, Thomas Dougherty published a report on the complete cure of cancerous tumors in mice after using PDT with a porphyrin derivative. This breakthrough result was the beginning of further research on the use of PDT in humans. Just three years later, Dougherty reported the successful use of PDT in the treatment of 113 skin tumors (primary and metastatic) in 25 patients. Of these cases, 98 tumors were completely cured, 13 showed a partial response to therapy, and only 2 tumors were completely resistant to therapy [15]. Despite very good therapeutic effects obtained using HpD, due to the long-lasting phototoxicity (which lasted from four to six weeks), the search for alternative photosensitizing compounds began. Among 1500 patients who underwent PDT with Photogem^®^, 91 percent showed a clear therapeutic effect and 62 percent of patients achieved complete tumor clearance. In the remaining patients, 29 percent had partial tumor shrinkage, and its reduction was at least half. For patients with a previous diagnosis, 92 percent showed complete tumor clearance [16].

The history of PDT is a story of humanity’s constant striving to overcome the limits to possibilities. It is a story that does not end with the achievements of 1990, which is, instead, when it gains real momentum. In the following decades, scientists not only developed this technique, but also discovered surprising new applications of light in medicine. In this work, we will look at how a seemingly simple beam of light became a powerful tool in the hands of doctors, enabling the fight against diseases that, until recently, seemed incurable. We will follow the evolution of PDT, which is proof that the human mind knows no limits when it comes to saving lives. The purpose of this work is to explore and present the evolution of PDT as a medical tool that has transformed a simple beam of light into a powerful tool to fight diseases that were previously considered incurable.

## 2. Materials and Methods

### 2.1. Literature Search

The literature search was a key step in developing the history of PDT. A systematic analysis of scientific works published until 2024 available on platforms dedicated to scientific papers was used. This process included a careful review of available articles, reviews, clinical trials, and literature reviews related to photodynamic therapy. The keywords used for the search were “PDT”, “photomedicine”, and “phototherapy”.

### 2.2. Inclusion and Exclusion Criteria

Inclusion criteria included research papers that focused on PDT both in the context of basic science and clinical applications. Articles containing information on new scientific discoveries, technological innovations and advances in the field were given priority. On the other hand, works that did not provide relevant data or were not related with the development of PDT were excluded from the analysis.

### 2.3. Analysis Process

After screening the papers in accordance with the inclusion and exclusion criteria, an in-depth analysis of the selected articles began. Key information was identified, such as stages of development of PDT, photosensitizers used, imaging techniques, and results of clinical trials. The analysis also included an assessment of the methodological quality of the studies examined.

### 2.4. Analysis Categories

When analyzing the literature across materials and methods, several key categories were identified. These included the following:

- History of PDT;
- Application of PDT in various fields of medicine;
- Scientific discoveries influencing the development of this method;
- Technological innovations and their impact on PDT effectiveness.

### 2.5. Systematization of Data

The collected information was systematized according to chronological order, illustrating the evolution of PDT over the years. Additionally, information was grouped thematically, which allowed the identification of key information areas of research and achievements. Carrying out the analysis of materials and methods in accordance with the described criteria allowed us to obtain a comprehensive and in-depth picture of the history of PDT and current scientific achievements in this field.

## 3. Results

### 3.1. Mechanism of Action of PDT

**Figure 2:**
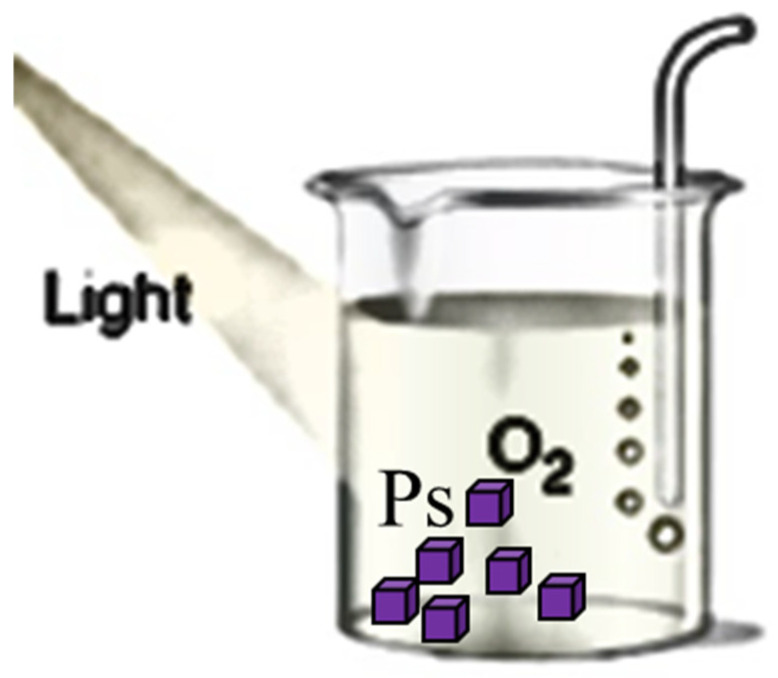
The generation of singlet oxygen in liquid solutions.

The three principal components of PDT are the PS, visible light, and oxygen. These individually harmless components, when used together, yield potent reactive oxygen species (ROS) (Figure 2). ROS are highly reactive chemicals formed from diatomic oxygen ($O_{2}$), water, and hydrogen peroxide. ROS comprise hydroperoxide ($O_{2}$H), superoxide ($O^{2-}$), hydroxyl radical (OH^.^), and singlet oxygen. Singlet oxygen, systematically named dioxygen(singlet) and dioxidene, is a gaseous inorganic chemical with the formula ^1^$O_{2}$. Singlet oxygen is the active species in PDT [17,18].

The formation of singlet oxygen at a specific biological site is extremely important for understanding the properties of tumor destruction by directed and concentrated singlet oxygen. Reactive products formed by the interaction with singlet oxygen give rise to the desired toxic effect. There are three goals that are key to the success of the PDT research: (1) to quantify singlet oxygen levels in solution, (2) to establish cytotoxic dose–response curves for humans, and (3) to determine the distance-related cytotoxic effects of singlet oxygen delivery.

**Figure 3:**
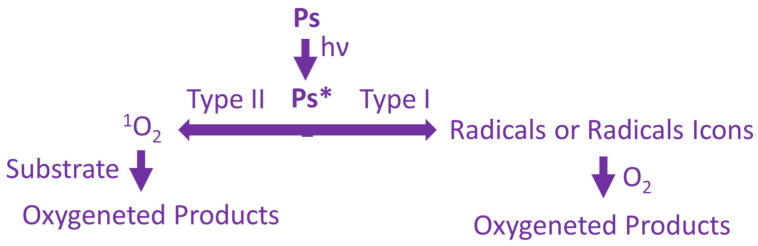
The mechanism of PDT actions. Ps*—excited state of PS.

The mechanism of action of PDT (Figure 3) includes two main processes:

1. Type I mechanism: The photosensitizer in the excited state transfers energy to biomolecules, creating free radicals and ROS that destroy cancer cells [19];
2. Type II mechanism: The PS transfers energy directly to oxygen, creating singlet oxygen, which has strong oxidizing properties and destroys cancer cells [20].

The effectiveness of PDT depends on the oxygen concentration, the structure of the PS, and the environmental conditions in the tissue. Photodamage leads to cell death through apoptosis (programmed cell death) or necrosis (cell death due to damage) [21,22,23]. Cell death results from many factors, e.g., the release of cytochrome c from damaged mitochondria. This is a signal for apoptosis. Singlet oxygen does not ‘destroy cancer cells’; it causes apoptosis, a death pathway. Autophagy is cytoprotective; it can recycle damaged mitochondria before they can release cytochrome c.

Most organic compounds are in the singlet ground state. However, oxygen is characterized by a triplet state (as the basis) and the excitation to singlet. Because of this fact, the excited PS particles do not damage organic cellular structures and react only with oxygen particles dissolved in the cytoplasm [23]. It is assumed that the type II mechanism is the most important process determining the effectiveness of PDT. However, the ratio of the contribution of both mechanisms depends on many factors, including the oxygen concentration, the tissue dielectric constant and pH, and the structure of the PS [24]. The mutual contribution of different types of cell death depends on the location of the PS within the cell. Damage to the mitochondria may lead to apoptosis, the destruction of cell membranes and loss of integrity may induce necrosis, and damage to the lysosomes or endoplasmic reticulum may provoke autophagy [25,26].

### 3.2. Development of PDT Technology

**Figure 4:**
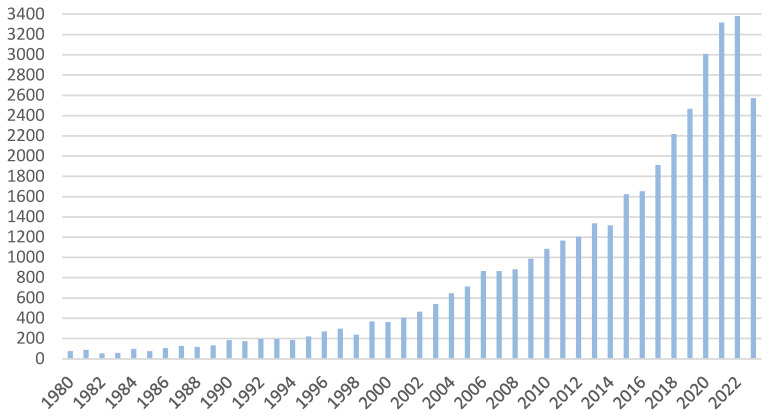
Number of published scientific papers on PDT according to Pubmed.

The development of PDT technology since 1980 has brought about numerous innovations and advances that have significantly improved the effectiveness and application of this method in medicine (Figure 4). The main aspects of PDT development include the selection of appropriate photosensitizing drugs, the improvement of light source technology, the adaptation of dosing protocols, the development of imaging, and the miniaturization of devices. Over the years, interest in PDT has grown at a dynamic pace, as evidenced by the number of scientific works published in the years 1980–2023. The smallest number of works written occurred in 1982 and amounted to 53 works, while their largest number in 2022 reached 3381. This shows how dynamically this field is developing and how much interest there is among scientists in this treatment method.

#### 3.2.1. Photodynamic Therapy in the Years 1990–1995

The first milestone in the history of PDT treatment (with Photofrin^®^) was achieved in 1993, when Canada became the first country in the world to approve this drug for the preventive treatment of bladder cancer. This event marked the beginning of Photofrin’s^®^ international expansion. Subsequently, approvals for Photofrin^®^ were obtained in the Netherlands and France. In these countries, the drug began to be used to treat advanced esophageal and lung cancer. Germany also recognized Photofrin’s^®^ potential, approving it for the treatment of early-stage lung cancer. Japan went a step further by approving Photofrin^®^ not only for the treatment of early-stage lung cancer, but also for esophageal, stomach, and cervical cancers, as well as cervical dysplasia.

In 1993, as a result of a study conducted in Canada, Photofrin-PDT was approved for prophylactic treatment after the surgical removal of papillary bladder tumors in patients at high risk of recurrence. Although the full results of this study have not been published, the preliminary results were presented in 1991 [27]. After one year of follow-up of 34 patients, disease recurrence was found in 81% of patients who did not receive PDT after surgery and in 39% of patients treated with PDT. The mean time to recurrence was 91 days for the control group and 394 days for the PDT-treated group. Among patients receiving PDT, one-third experienced photosensitivity and 93% had urinary symptoms. Due to the severe and long-lasting side effects, Nseyo and colleagues [28] suggested the use of multiple therapies at a lower drug dose to reduce the frequency and severity of symptoms after PDT for superficial bladder cancer.

In December 1995, after the completion of phase three clinical trials in the United States, Photofrin^®^ received FDA approval [29]. A multi-center study compared PDT with Nd-YAG laser thermal ablation in the treatment of partially obstructive esophageal cancer. The study included 236 patients and showed similar reductions in dysphagia in both groups. However, PDT showed a longer tumor response time (32% at 1 month vs. 20% for Nd-YAG) and more complete responses (negative endoscopic biopsies) than Nd-YAG (9 vs. 2). In some subgroups, PDT showed higher objective responses than Nd-YAG, especially in the upper and lower third of the esophagus, and also for tumors larger than 10 cm. However, the number of patients in these groups was too small to achieve statistical significance. Fewer procedures were required for PDT (mean 1.5) than for Nd-YAG (2.4), and the median survival was the same for both groups. There were more adverse reactions in the PDT group (92%) than in the Nd-YAG group (82%), but the rate of withdrawal from the study due to an adverse reaction was similar in both groups. Significantly, more esophageal perforations occurred in the Nd-YAG group (7%) than in the PDT group (1%). Sunburn reactions occurred only in the PDT group (19%) and were all mild. The effectiveness of both treatments was equivalent; serious adverse reactions occurred at the same rate in both treatments, except for a higher incidence of perforation in the Nd-YAG treatment. PDT was considered more comfortable for the patient, was easier to perform than Nd-YAG ablation, and was particularly beneficial in situations where Nd-YAG is difficult to perform due to the tumor morphology or location.

#### 3.2.2. Photodynamic Therapy in the Years 1996–2000

In 1996, Biel [30] published the results of a study on the treatment of early-stage head and neck cancer with Photofrin^®^. This study included a variety of tumor types, including 29 patients with laryngeal cancer (22 of which were superficial), 32 patients with nasopharyngeal cancer, 1 patient with nasal cavity cancer, 2 patients with Kaposi’s sarcoma of the palate, 3 patients with nasopharyngeal cancer, and 5 patients with laryngotracheal papilloma. Patients received a dose of 2.0 mg/kg Photofrin^®^ and, 48 h later, were irradiated with 630 nm light using a microlens fiber at 50–75 J/cm^2^. For tumors larger than 3 cm, diffusion fibers were implanted, and a dose of 100 J/cm fiber was delivered interstitially. All 22 patients with superficial laryngeal cancer achieved a complete response, with a mean follow-up of 30 months (up to a maximum of 67 months). Similar results were achieved in patients with oral, intranasal, or nasopharyngeal cancer who were followed for a mean of 33 months (up to a maximum of 61 months). Five patients with recurrent laryngeal/tracheal papilloma initially responded to PDT, but disease recurrence was observed 6 months after PDT. Two patients required oral steroids for 5 days due to sunburn. Post-treatment pain ranged from mild to severe but was effectively controlled with oral analgesics.

In 1997, researchers conducted the largest PDT study using Photofrin^®^ on patients with superficial esophageal cancer [31]. This disease often occurs together with Barrett’s esophagus, a condition in which the squamous epithelium of the esophagus is replaced by the glandular epithelium of the stomach due to acid reflux. Patients with Barrett’s esophagus are at risk of developing esophageal cancer, for which the standard treatment is esophagectomy, a surgical procedure with a high mortality rate. The study included 55 patients and, after six months of follow-up after PDT, showed that 24 of 36 patients with initial high-grade dysplasia and Barrett’s esophagus had no dysplasia and 7 had no residual Barrett’s esophagus. Of 36 patients with high-grade dysplasia, 3 did not respond to treatment and 9 converted to low-grade dysplasia. The technique involved injecting 2.0 mg/kg Photofrin^®^, followed by light delivery 48 h later. In some patients, a 3, 5, or 7 cm balloon catheter was used, in which the light delivery fiber with a diffuser of the appropriate length was centered. The balloon allowed for the proper dilation of the esophagus and provided uniform light delivery to the affected areas. Complications included esophageal stricture in 29 patients, which required dilation. The photosensitivity was of low frequency. For PDT compared with surgery, the mortality rates were 0% and 6–14%, respectively. Moreover, PDT was an outpatient procedure with a much shorter recovery time and was associated with much lower procedural costs.

In 1999, a new era began in the United States in the treatment of age-related macular degeneration (AMD), which is the leading cause of irreversible vision loss in people over 50 years of age. This year, PDT with verteporfin (Visudyne^®^) was approved by the Food and Drug Administration (FDA) for the treatment of patients with the predominantly classic form of subfoveal neovascularization, which is one of the more serious types of AMD. The results from phase I–II clinical trials showed that PDT with verteporfin could safely stabilize choroidal neovascularization (CNV) leakage in most patients for up to 3 months. Phase III trials that assessed the long-term prognosis of the PDT treatment of CNV in AMD were successful, and several tens of thousands of patients were successfully treated [32].

Moreover, in 1999, the USA saw a groundbreaking use of PDT in dermatology. PDT has gained recognition for its effects in the treatment of solar keratoses—one of the most common skin lesions [33]. These keratoses are characterized as erythematous, flat, and scaly lesions, appearing mainly on sun-exposed areas of the skin, especially in people with fair skin. Their diameter may vary from a few millimeters to several centimeters [34]. Traditionally, solar keratosis has been treated with methods such as cryosurgery, laser ablation, dermabrasion, and other techniques. However, the introduction of 5-aminolevulinic-acid-based PDT (ALA-PDT) and methyl-aminolevulinate-based PDT (MAL-PDT) into clinical practice has yielded breakthrough results. The approval of these treatment methods resulted in high effectiveness, achieving a 89–92% elimination of skin lesions, especially those located on the face and scalp [35].

#### 3.2.3. Photodynamic Therapy in 2001–2005

Temoporfin, also known as m-tetrahydroxyphenylchlorin (mTHPC), is an active substance used in photodynamic therapy (PDT). In 2001, the medicinal product Foscan^®^, containing temoporfin, was approved by the European Medicines Agency (EMA) for the treatment of advanced squamous cell carcinoma of the head and neck. In 2000, it was submitted to the US Food and Drug Administration (FDA), but it was not approved there. Temoporfin (Foscan^®^) has been studied in various clinical contexts, including the treatment of unresectable cholangiocarcinoma, unresectable non-small-cell lung cancer, and nasopharyngeal cancer. Initially, the medicinal product Foscan^®^ was produced by Scotia Pharmaceuticals in Scotland, but, later, the rights were taken over by the German company Biolitec. Although Foscan^®^ has many advantages and a high absorption peak at 652 nm, it also has some disadvantages. Due to its exceptional potency, it can damage healthy tissue surrounding the tumor. Furthermore, there is a risk of skin burns due to photosensitizer extravasation at the infusion site [36].

In 2003, another study was conducted on PDT using verteporfin. This study, which was non-randomized and prospective, included 19 patients suffering from symptomatic, limited angiocellular hemangioma. Before starting PDT, four patients underwent unsuccessful initial treatment, such as external beam irradiation or laser photocoagulation. The eligibility criteria for the study included the presence of subretinal exudation involving the macula, decreased visual function, and additional symptoms such as metamorphopsia. During PDT, verteporfin was used at a concentration of 6 mg/m^2^ of the body surface and a light dose of 100 J/cm^2^ with a wavelength of 692 nm. The average follow-up time of patients was 10.6 months, and the number of treatment sessions ranged from 1 to 5. The results were promising. Vision improved by at least one line in 73.3% of patients and by at least two lines in 42.1% of patients. Exudation completely resolved in 94.8% of cases, and tumor size decreased in all tumors examined. No recurrences or local or systemic side effects were observed during the follow-up period [37].

In 2004, there were reports of clinical trials using PDT in the treatment of cholangiocarcinoma (CC). In these studies, almost two-thirds of patients died from progressive CC. Additionally, approximately 5 to 10% of patients died from a paraneoplastic pulmonary embolism associated with an advanced tumor stage or from gastrointestinal bleeding secondary to tumor invasion into the duodenum or biliary cirrhosis. One quarter of patients died prematurely due to severe infectious complications. Chronic cholangitis led to secondary biliary cirrhosis and fatal complications such as variceal bleeding in two patients with a slow tumor progression. Studies have shown that, when PDT was used repeatedly to treat progressive disease and the resulting segmental biliary tree obstructions, a significant increase in median survival could be expected in the range of >9 to 16.2 months. Even patients in poor condition benefited from PDT [38]. In 2005, scientists from the Weizmann Institute in Israel, Avigdor Scherz and Yoram Salomon, made a breakthrough in the development of Tookad^®^, a drug used in photodynamic therapy. Struggling with the problems associated with the use in clinical trials, they developed a new, water-soluble derivative of Tookad^®^. They used a process known as aminolysis, using the amino acid taurine, to create a new compound, which was named WST-11. This compound, later named Stakel^®^ and then Padeliporfin^®^, proved to be a key step forward in photodynamic therapy. The production of this new compound was undertaken by Steba Biotech based in Luxembourg. WST-11, like its predecessor WST-09, was found to act very rapidly and, upon irradiation, causes vascular shutdown, which is characteristic of a type I photochemical process called vascular-targeted PDT or VTP [39].

#### 3.2.4. Photodynamic Therapy in 2006–2010

In 2006, it was announced that PDT may be effective in the treatment of lung cancer. Studies have shown that PDT with Photofrin^®^ increases the expression of vascular endothelial growth factor (VEGF) and prostaglandin E2 in murine tumors. Additionally, it was noticed that the combination of VEGF or cyclooxygenase-2 inhibitors with PDT therapy increases its therapeutic effectiveness. It has also been found that tumors treated with PDT show increased the expression of matrix metalloproteinases (MMPs), and the pharmacological inhibition of MMPs may enhance the antitumor effects of PDT in vivo [40].

In 2007, PDT gained importance not only in the fields of dermatology and oncology, but also in microbiology. Research conducted by Smijs’ team used an ex vivo human skin model to assess the ability of porphyrins to eliminate *T. rubrum*. PSs in liquid carriers were applied to skin that had previously been infected with a dermatophyte. It was found that short incubation periods (8 h) led to the complete destruction of the fungus after irradiation (108 J cm^−2^, 580–870 nm). However, longer incubation periods (>24 h) before exposure did not lead to the elimination of the fungus [41].

At the same time, PDT has been intensively researched in various medical fields. In one study conducted by a team of scientists [42], 15 patients with histologically confirmed actinic cheilitis were treated with PDT using MAL. After two treatment sessions, one week apart, complete clinical remission was observed in almost half of the patients. Nevertheless, histopathological examinations showed signs of dysplasia in most patients, which could suggest that the uneven surface of the lips could lead to the uneven absorption of the photosensitizing agent.

Additionally, in a European, randomized, multicenter, placebo-controlled trial [43], the effectiveness of PDT with MAL was compared with cryotherapy and 5-FU in patients with Bowen’s disease. The results of this study indicated that PDT with MAL achieved the highest rate of complete remission at 12 months.

Moreover, research conducted by Zane and colleagues [44] showed that PDT can affect collagen fibers, which suggests the possibility of stimulating collagen synthesis. These studies have also shown that PDT can lead to the reorganization or accumulation of new collagen fibers, which may help to improve the skin texture.

In 2009, PDT was described as a potentially effective method for the local treatment of nasopharyngeal cancer (NPC), which is not associated with the severe side effects observed in radiotherapy. PDT is effective in eliminating NPC, with the effective local inhibition of tumor growth. Many advantages of PDT have been noted, including the possibility of repeating the therapy, the lack of cumulative effects, and specific effects on cancer cells. The studies used a first-generation PS, hematoporphyrin. Others, in turn, have shown that the second-generation PS, temoporfin, is more effective. Nasopharyngeal illumination was a challenge, so a new dedicated light delivery applicator was developed [45]. In 2010, a study was conducted on the use of PDT in the treatment of anal cancer [46]. All study participants went through the PDT process in accordance with the guidelines, without any harm to their health. The procedure was well-tolerated with no unforeseen or excessive complications. The entire therapy was performed on an outpatient basis, without the need for hospitalization or visits to the emergency department. No complications were observed after the administration of Photofrin^®^ and none of the patients experienced solar photosensitization. Immediately after PDT treatment, the tumor area became dark and red and began to secrete fluid. Interestingly, none of the patients reported pain at this point. After 24 h, the tumor area remained dark but was no longer shedding fluid. Healthy tissue appeared to be clinically unchanged compared to unilluminated regions. The treatment sites were very sensitive to touch. Similar results were observed after 48 h. All patients controlled their sphincters during this time. Approximately 6–12 h after PDT, patients experienced an increase in pain around the anus, which was most noticeable during bowel movements and prolonged sitting. By using additional painkillers, they were able to minimize these symptoms. The intensity of this discomfort decreased significantly after 72–96 h in all cases. Applying ice to the anal area provided excellent and rapid short-term relief. The use of analgesics was sporadic from the end of the first week and was not necessary 1 month after PDT. Patients were then followed monthly or less frequently. At the first follow-up visit one month later, the tumor was necrotic and appeared to be healing peripherally. No patient had significant pain at this point, and all returned to normal follow-up examinations. Each patient had a repeat biopsy between 3 and 4 months after PDT, and all showed no evidence of disease (NED). After 6 months, all patients were re-evaluated. None showed local or pelvic recurrence, but two patients had evidence of systemic disease and received chemotherapy. At the last follow-up visit (18–48 months), no patient had local failure, wound healing problems, or sphincter damage caused by PDT [46].

#### 3.2.5. Photodynamic Therapy in 2011–2015

In 2011, a pioneering study was conducted that opened new horizons in the treatment of potentially malignant oral diseases using PDT. In this prospective study, a group of 147 patients underwent PDT with surface illumination using 5-ALA or meta-tetra(hydroxyphenyl)chlorin (mTHPC) as PS. The patients, with an average age of 53, did not experience any complications after the procedure. During an average of 7.3 years of follow-up, clinical and histopathological features as well as the rate of recurrence and malignant transformation were compared. The analysis showed that homogeneous leukoplakia occurred in 55 patients, heterogeneous leukoplakia in 73 patients, and 19 patients had erythroplakia. The patients were dominated by former and current lifelong smokers (84.4%). Erythroplakias have mainly been identified in heavy lifelong smokers. The most frequently identified primary anatomical locations are the lateral edge of the tongue, the floor of the mouth and the retromandibular area. Among the patients, 33 were identified as having moderate dysplasia, while 63 patients had severe dysplasia, and 32 patients had a histopathological diagnosis of carcinoma in situ. The recurrence rate for laser surgery was approximately 11.6%. Malignant transformation was observed in 11 patients (7.5%), on the tongue, floor of the mouth, and retromandibular area. Recurrence and malignant transformation were mainly identified in erythroplakias and heterogeneous leukoplakias. In conclusion, a 2011 study showed that 5-ALA-PDT and/or mTHPC-PDT offer an effective alternative therapy for potentially malignant oral diseases. A complete response to therapy was identified in 119/147 patients (81%) [47].

Moreover, in 2011, a significant study was conducted to understand the effects of PDT on papilloma acuminata. The study involved 15 people with histologically confirmed, treatment-resistant acuminal papilloma located in various locations. Patients underwent several cycles of PDT after receiving ALA. The study showed complete recovery in nine out of fifteen people after five PDT sessions. Lesions in the anal area showed particularly rapid remission. As progress towards the complete removal of lesions was observed, the dense infiltration of CD4+ T cells was observed in the superficial skin, accompanied by the accumulation of Langerhans cells. At the same time, CD8 began to increase in the lesions of patients responding to treatment, and Langerhans cells appeared to migrate toward the dermis. CD68+ macrophages apparently did not participate in the immune response. In summary, the 2011 study provided valuable information on the effect of ALA-PDT on infiltrating immune cells in acuminal papillomas. The results confirmed previous clinical data, suggesting that the rapid activation of specific immunity in the affected skin, CD4+ T cells, and dendritic cells could be responsible for healing. This study represented an important step in understanding and treating; unlike previous studies that focused on the use of red PDT light after long-term incubation and occlusion, this study focused on the use of blue light and short-term incubation without occlusion. The study included adults with at least two resistant warts on the back of the hand or periungual warts. Patients were randomly assigned to receive the placebo or 20% ALA. After a 1 h incubation period, the papillae were exposed to 417 nm blue light for 1000 s. The overall response pattern was not statistically different between the two groups. Only two of the 12 warts treated with cryotherapy disappeared. This response pattern was not statistically different from PDT-treated warts. One patient complained of a burning sensation after ALA application, which was attributed to the acidic solution penetrating through the nipple cracks [48].

In 2013, a study was conducted on the treatment of peri-implantitis, a bacterial complication following the implantation of dental implants. This study was randomized, comparative, and included 20 patients and 20 controls. The aim was to compare the antibacterial effectiveness of PDT with surgical therapy in patients with peri-implantitis who received dental implants with a rough surface. The surgical group underwent mucoperiosteal flap surgery with scaling on the implant surfaces and the removal of granulation tissue. Microbiological testing was assessed before and after interventional treatment, at weeks 12 and 24 in the subjects. No significant difference in total anaerobic bacteria was observed between patients treated with PDT and those treated surgically. However, PDT was associated with a significant decrease in bleeding rates and inflammatory secretions, visible even 24 weeks after the procedure [49].

In 2015, a study was conducted on the treatment of actin damage, which is responsible for the development of multiple, recurrent non-melanoma skin cancers (NMSCs), including actinic keratoses (AK). This study compared the effectiveness and safety of PDT with MAL and imiquimod cream (IMIQ) 5% in preventing the development of new NMSCs in patients with field lesions. The study included patients with a tumor field on the face or scalp who were randomly assigned to receive MAL-PDT on one side and IMIQ 5% on the mirror field. The main aim of the study was to examine the number of new lesions in the treated fields during a 12-month observation period. No significant difference in the development of new NMSCs was observed between patients treated with MAL-PDT and those treated with IMIQ. Both treatments were safe and well-tolerated. Patients showed a preference for MAL-PDT based on the procedure, response rates, and future choice. In summary, both MAL-PDT and IMIQ 5% are safe and well-tolerated treatments that effectively prevent the development of new AKs in patients with tumorigenesis. However, the MAL-PDT treatment seems to be preferred by patients [50].

#### 3.2.6. Photodynamic Therapy in 2016–2020

In 2016, a study was conducted to assess the safety of PDT using 3-(1′-hexyloxyethyl) pyropheophorbide-a (HPPH) in the treatment of early stages of laryngeal diseases. This study, conducted in a single center, was open-label and non-comparative. It included patients at high risk for dysplasia, carcinoma in situ, and T1 squamous cell carcinoma (SCC) of the larynx. The primary aim of the study was to determine the safety of the therapy and the maximum tolerated dose (MTD), and the secondary aim was to assess the response to treatment. In total, 29 patients participated in the study and 30 lesions were treated. The most common adverse event was transient hoarseness. The most serious adverse event was severe edema requiring tracheostomy, which occurred in two patients within hours of therapy. The maximum tolerated dose was 100 J/cm^2^. Patients with T1 squamous cell carcinoma showed a good overall response (82%) to HPPH-PDT therapy at the maximum tolerated dose. The conclusions of the study indicate that HPPH-PDT therapy can be safely used in the treatment of early laryngeal cancer, which suggests its potential effectiveness [51].

Moreover, in 2016, a study was conducted to investigate the effectiveness and safety of PDT using hemoporfin and a 532 nm laser in the treatment of a port-wine stain. This study, carried out in eight hospitals in China, included patients aged 14 to 65 years affected by this mole. In the first phase of the study (day 1 to week 8), patients were randomly assigned to treatment. The treatment group received 532 nm laser irradiation (96–120 J/cm^2^) with hemoporfin (5 mg/kg; PDT-hemoporfin, n = 330), while the placebo group received irradiation with placebo (PDT-placebo, n = 110). In the second phase of the study (from weeks 8 to 16), all patients were offered treatment. The main goal of the study was to determine how many patients achieved at least some improvement at week 8. Additionally, we assessed how many patients achieved the almost complete relief of symptoms or at least a major improvement at week 8, and how many patients achieved early complete relief, at least a major improvement, or at least some improvement at week 16. The investigator and patient satisfaction with the treatment was also examined at weeks 8 and 16. The study results showed that the PDT-hemoporfin treatment group achieved significantly better results compared to the placebo group. Nearly 90% of patients in the treatment group achieved at least some improvement at week 8. All secondary efficacy endpoints were also higher in the treatment group. Treatment reactions occurred in almost all patients in the treatment group, and hyperpigmentation occurred in approximately 23 of 100 treated patients. The results of the study allowed us to suggest that PDT using hemoporfin is an effective and safe method of treating a port-wine stain in patients of different ages [52].

In 2017, a study was conducted focusing on the chronic and incurable disease lichen sclerosus of the vulva, which causes various unpleasant symptoms and serious consequences. The aim of this study was to investigate the effectiveness of PDT in the treatment of this disease. The study included 102 patients aged 19 to 85 who suffered from vulvar lichen sclerosus. All patients underwent PDT, which used 5% 5-ALA in the form of a gel. The affected areas were irradiated with a PhotoDyn 501 halogen lamp (590–760 nm) during a ten-minute treatment. Treatments were repeated weekly for 10 weeks. The results of the study were promising. PDT showed good therapeutic effects, with an 87.25% improvement rate in patients suffering from lichen sclerosus. The greatest improvement was observed in the reduction in subepithelial petechiae and telangiectasia (78.95%), as well as in the reduction in erosion and cracks (70.97%). The partial resolution of lichenization with hyperkeratosis was observed in 51.61% of cases. The smallest improvement was observed in the reduction in atrophic changes (an improvement in 37.36% of cases). These results allowed us to conclude that PDT has excellent effects in the treatment of lichen sclerosus and, additionally, gives very good cosmetic effects [53].

In 2018, a study was conducted on rosacea, a common chronic skin disease that presents with redness, erythema, pustules, and telangiectasia. Due to the tendency of the disease to relapse, a multi-faceted approach to its control is necessary. This study aimed to investigate the effectiveness and safety of PDT using ALA for the treatment of rosacea in Chinese patients with Fitzpatrick skin types III and IV. The study included 20 patients with rosacea, both erythematotelangiectatic and papulopustular types. 5-ALA was used in the form of an oil-in-water emulsion, which was applied to the lesions under occlusion with plastic foil for 2 h. Then, the lesions were irradiated with red LED light with a power of 100 mW/cm^2^, 80–90 J/cm^2^, for 15 min in each session. The treatments were repeated every 10 days for 10 weeks. The study results were promising. All patients showed gradual improvement compared to baseline. All clinical inflammatory changes completely disappeared after 24 weeks. Subjective symptoms such as redness, itching, stinging, burning, etc. disappeared and did not recur during the follow-up period. The main side effects are pain, redness, swelling, and post-inflammatory hyperpigmentation. All side effects were transient and tolerable in all patients. To sum up, the results of the study showed that photodynamic therapy with ALA is an effective and safe method of treating erythematotelangiectatic or papulopustular rosacea [54].

In 2019, a pioneering study was conducted to investigate the potential benefits of the concomitant use of PDT as a tumor ablation method in combination with minimally invasive stabilization procedures for pathological vertebral compression fractures (VCFs), such as vertebroplasty (VP) and balloon kyphoplasty (KP). This study aimed to investigate whether PDT could complement the improvement in mechanical stability provided by vertebral cement augmentation (VCA). The study included thirty patients with various primary tumors who were treated with PDT and VP or KP. The study used a single dose of 6 mg/m^2^ of the clinical photosensitizing agent Visudyne^®^ with increasing doses of laser light. After a light-only control group (n = 6), subsequent drug and light treatment groups (n = 6 each) received 50, 100, 150, and 200 J/cm. Cement augmentation of the vertebrae was performed within 15 min after PDT. Patients were clinically evaluated at 1 and 6 weeks. The main evaluation criterion was safety from a neurological perspective. The study results were promising. All patients underwent a vertebral PDT procedure, which was technically feasible and provided to all patients in the study. Neither dose group showed a significant increase in pain as defined by the generic Short Form-36 (SF-36), as well as the disease-specific European Organisation for Research and Treatment of Cancer—Quality of Life Questionnaire—Bone Metastases 22 (EORTC-QLQ-BM22) and European Organisation for Research and Treatment of Cancer—Quality of Life Questionnaire—Core 15—Palliative (EORTC-QLQ-C15-PAL) questionnaires. The 50 and 100 J/cm groups showed the most significant pain reduction (*p* < 0.05). It was found that 12 (40%) patients experienced complications during the study, including 3 patients with the further progression of vertebral fracture after 6 weeks despite VCA. No complications were directly attributable to PDT. In summary, the study results suggested that vertebral PDT as an adjunct to VCA is safe from a pharmaceutical and neurological point of view. The results of this study motivate a larger-scale study to evaluate the potential effectiveness of PDT in the treatment of vertebral metastases [55].

In 2019, a study was conducted to assess the clinical and microbiological periodontal parameters after the use of additional antibacterial photodynamic therapy (APDT) in HIV-infected and -uninfected patients suffering from necrotizing ulcerative periodontitis (NUP).

The study included HIV-infected patients (group I) and healthy patients (group II) suffering from NUP. Patients were randomly divided into two groups that underwent APDT and scaling and root planning (SRP), respectively. Clinical periodontal parameters such as the full dental plaque index (FMPI), bleeding on probing (FMBOP), probing depth (PD), and clinical attachment level gain (CAL) were examined. The levels of bacteria, including *Aggregatibacter actinomycetem-comitans* (Aa), *Porphyromonas gingivalis* (Pg), and *Tannerella forsythia* (Tf), were assessed by PCR. All assessments were performed at baseline, 3 months, and 6 months. The study results showed that all periodontal parameters, including FMPI, FMBOP, PD, and CAL, significantly improved in both HIV-infected and uninfected patients. The reduction in mean PD was higher only after APDT treatment among patients in group II compared to patients in group I during follow-up (*p* < 0.05). The gain in mean CAL was higher only after APDT treatment among patients in groups I and II during follow-up (*p* < 0.05). All bacterial levels decreased from baseline to follow-up after both APDT and SRP treatment in both groups (*p* < 0.05). APDT showed significantly reduced Aa and Tf levels at 3 months and only Aa at 6 months among HIV-positive patients, while Pg and Tf levels were significantly reduced at 3 months and only Aa at 6 months in HIV-negative patients (*p* < 0.05). In summary, the use of antibacterial photodynamic therapy as an adjunct to scaling and root planning has been effective in improving clinical periodontal parameters and bacterial levels in HIV-infected patients suffering from NUP. However, the improvement was not greater compared to HIV-uninfected patients [56].

In 2020, a prospective, randomized, self-controlled study was conducted to evaluate the effectiveness, pain, and safety of the modified photodynamic therapy (M-PDT) in the treatment of genital warts. Papillae were randomly assigned to the M-PDT or coherent photodynamic therapy (C-PDT) side. 5-ALA; 20% was incubated for 3 h before the exposure of patients to red LED light (100 J/cm^2^) on the C-PDT side and for 30 min before exposure to red LED light (300 J/cm^2^) on the side M-PDT. Therapy was administered weekly for three weeks. Cure rates were determined at 1 week and recurrence rates at 4, 8, and 12 weeks after treatment. Pain and other side effects were also studied. A total of 24 patients with genital warts participated in the study. Twenty patients completed the study. The cure rates were 98.17 ± 3% on the M-PDT side and 98.20 ± 6% on the C-PDT side (*p* > 0.05). The recurrence rates were 11.11 ± 3% and 10.53 ± 4% (*p* > 0.05). However, M-PDT was almost painless (mean score 0.3 ± 0.47, range 0∼1), which was much less than that of the C-PDT side (mean score 3.6 ± 0.94, range 0∼1) (*p* < 0.05). Local redness, mild swelling, and erosion were observed on both sides. The results of the study established that the modified photodynamic therapy is virtually painless and has similar effectiveness to the conventional photodynamic therapy. This is a significant breakthrough in pain management in PDT [57].

#### 3.2.7. Photodynamic Therapy in 2021–2023

In 2021, another human wart treatment study was conducted and showed significant results. This study, a prospective, randomized, controlled experiment, included eighty patients with warts who were divided into three groups. Group A, consisting of 30 patients, underwent PDT sessions using endocrine methylene blue (MB) and intense pulsed light (IPL). Group B, also consisting of 30 patients, received only IPL sessions. Group C served as the control group. The response to treatment was assessed based on the clinical and dermatoscopic scores, cure rate, and ImageJ analysis, which included the wart surface and hemorrhagic structures or vessels. In group A, subjected to MB/IPL/PDT therapy, the clinical and dermatoscopic removal of warts was achieved in 43.3% of patients, which translated into a cure rate of 40.9%. In group B, which received only IPL sessions, these rates were 20% and 23.4%, respectively. The ImageJ analysis showed a greater reduction in the area of warts and hemorrhagic or vascular structures in group A. In conclusion, MB/IPL/PDT therapy proved to be an effective treatment option for warts, achieving a success rate of approximately 40% based on clinical and dermatoscopic evaluation. This efficiency was even higher using ImageJ analysis, which took into account both the wart surface and the surface of vessels and hemorrhagic dots. The latter were more affected by treatment [58].

In 2021, a randomized, controlled clinical trial was conducted to compare the effectiveness of PDT and the use of trichloroacetic acid (TAA) in the treatment of HPV warts around the anus and vulva. This study was conducted at the Women’s Health Outpatient Clinic in the city of São Carlos, São Paulo State, Brazil. The study included 36 patients, 31 of whom met the study requirements. Patients were randomly assigned to one of two treatment regimens: PDT or TAA. The PDT protocol used the prodrug MAL, which was incubated for 3 h and then irradiated at a wavelength of 630 nm (100 J/cm^2^). For TAA, the warts were gently soaked in acid using a cotton swab. Both treatments were repeated weekly until the lesions disappeared completely or until 10 sessions were performed. The main criterion for assessing the effectiveness of treatment was clinical analysis, and patients were followed up for 12 to 30 months after the end of treatment. Among 16 patients treated with PDT and 15 patients treated with TAA, the overall effectiveness was 63% and 60%, respectively. The recurrence rate was 0% for PDT and 33% for TAA. In summary, PDT not only successfully treated warts by physically destroying lesions and clinical lesions, but also appeared to modulate the immune system and/or reduce the local viral load, suggesting a lower recurrence rate compared to the TAA-treated group [59].

A 2022 study aimed to evaluate the effect of antimicrobial PDT (aPDT) as an adjunct to topical antiviral therapy in children with herpetic gingivostomatitis. The study involved 45 people aged 12 to 18 years who suffered from herpetic gingivostomatitis (HG). The subjects were divided into three groups depending on the type of treatment used. Group A consisted of 14 people (mean age 17.0 years) who received topical antiviral therapy (TAT). Group B included 15 people (mean age 17.7 years) who underwent aPDT. Group C included 16 people (mean age 18.0 years) who received local antiviral therapy with the addition of aPDT. Pain was assessed using a visual analogue scale (VAS) and the McGill Pain Questionnaire (MPQ), and HSV-1 quantification was performed. Proinflammatory cytokine levels, including interleukin 6 (IL-6) and tumor necrosis factor-alpha (TNF-α), were calculated using ELISA. The analysis of data obtained after the clinical assessment showed that all three groups experienced decreases in pain scores, HSV-1 loads, and pro-inflammatory cytokine levels. However, Group C (TAT + aPDT) showed a statistically significant improvement in the observed parameters compared to Group A (TAT) and Group B (aPDT) [60].

A study conducted in 2022 aimed to use PDT using 5-ALA in the treatment of nicotine stomatitis in smokers. The study involved 24 patients with this disease, who were divided into two groups: test (n = 12) and control (n = 12). Patients in the test group were treated with PDT using 5-ALA, while patients in the control group were advised to stop smoking for the duration of the study. PDT treatment was repeated on days 3, 7, and 14, and participants were then monitored at follow-up visits 4, 6, and 8 weeks after completion of treatment. Data analysis was performed using Statistical Package for the Social Sciences (SPSS) version 22.0. According to the results, patients in the test group showed statistically significant improvement at all three time points (*p* < 0.0001). A similar trend was also observed in the control group (*p* < 0.001), but the difference between both groups was significant. The results of this clinical study suggest that the use of PDT with 5-ALA can effectively reduce the clinical symptoms of nicotine stomatitis without negative side effects. Therefore, PDT with 5-ALA seems to be a promising therapeutic option, especially when combined with smoking cessation [61].

A study conducted in 2023 aimed to compare two methods of nasal decolonization in patients undergoing chronic hemodialysis who were carriers of Staphylococcus aureus. Infections in which *S. aureus* is the etiological agent constitute a significant health problem among this group of patients, and nasal colonization with this pathogen increases the risk of infection. The study used two decolonization approaches: PDT and mupirocin treatment. PDT, which does not induce antibiotic resistance, consisted of a single application of light with a wavelength of 660 nm (400 mW/cm^2^, 300 s) using 0.01% methylene blue as a PS. In turn, mupirocin treatment included the use of this antibiotic topically twice a day for 5 days. The study results showed that both methods were effective in eliminating *S. aureus* from the nose immediately after treatment. However, within 3 months after completion of PDT, 67% of patients who had negative cultures immediately after the completion of treatment were recolonized. No adverse events were reported in the PDT group. This study is an important step in the search for alternative methods of nasal decolonization in patients undergoing chronic hemodialysis. The study also indicated that larger studies are needed in the future to determine whether PDT is equivalent to the standard of care with mupirocin [62].

A scientific study conducted in 2023 focused on analyzing the therapeutic potential of PDT with indocyanine green (ICG-PDT) in the treatment of keloids, which are a common dermatological problem. As part of the study, patients with keloids were divided into four groups: control, photothermal therapy, PDT, and combined therapy. The aim of the in vitro study was to understand the mechanism of action of PDT in the context of keloid treatment, which could contribute to the optimization of its clinical application. The study results showed that ICG-PDT effectively inhibited the cellular activity and migration of keloid fibroblasts, and this effect was most visible when the photodynamic mechanism was operating. Additionally, in this experimental group, the induction of autophagy and apoptosis and the inhibition of collagen synthesis were observed. Moreover, these therapeutic effects could be achieved at relatively low drug concentrations [63].

A study was also published in 2023, which was a significant contribution to the current research on SARS-CoV-2. This study focused on evaluating the effectiveness of intranasal PDT in the context of SARS-CoV-2 infection. The main aim of the study was to investigate the effect of PDT on shortening the infectious period in SARS-CoV-2 carriers with mild symptoms. Additionally, this study focused on the analysis of SARS-CoV-2-specific effects of immune system stimulation and the safety of therapy. The study was conducted as a randomized, placebo-controlled clinical trial. Patients with a positive SARS-CoV-2 PCR within the previous 48 h were recruited and randomly assigned to receive PDT or the placebo. Patients with pneumonia were excluded from the study. The primary outcome of the study was a reduction in in vitro infectivity of nasopharyngeal samples on days 3 and 7 after the initiation of therapy. Additional results included the safety assessment and quantification of humoral and cellular immune responses. The study results indicate that intranasal PDT is safe in mildly symptomatic COVID-19 patients, reduces SARS-CoV-2 infectivity, and slows the decline in specific immune responses to SARS-CoV-2. CoV-2. These results constitute an important contribution to ongoing research on SARS-CoV-2 and may have a significant impact on future treatment strategies [64].

### 3.3. PDT in Clinical Treatment

#### 3.3.1. Head and Neck Tumor

Head and neck cancer was the seventh most commonly diagnosed cancer worldwide in 2018, with 890,000 new cases and 450,000 deaths, accounting for 3% of all cancers in the United States and more than 1.5% of cancer deaths. Although the incidence of head and neck cancer related to tobacco and alcohol is gradually declining worldwide, the incidence of HPV-related oropharyngeal cancer is increasing in North America and northern Europe, particularly among younger individuals [65].

PDT has been used effectively in cancers of the neck and head in the treatment of neoplastic lesions in the oral cavity, pharynx, and larynx, achieving satisfactory treatment effects while maintaining speech and swallowing functions. The most valuable study in this regard was conducted by Biel and his research team, when cancers of the neck and head were treated, mainly squamous cell carcinomas of the oral cavity, pharynx, and larynx, and cases of Kaposi’s sarcoma or melanoma were also treated in a group of over 300 patients. The treatment was based on the use of PDT with Photofrin^®^. In the study group of 133 patients with recurrent or primary laryngeal cancers carcinoma in situ (CIS), T1N0 and T2N0, who were treated with PDT, a 90% cure rate was noted after 5 years of monitoring, with an average follow-up time of 96 months. In the second group, consisting of 138 patients with squamous cell carcinomas of the oral cavity CIS and T1N0, PDT treatment was also used. Patients were monitored for up to 211 months, and, after 5 years, all patients achieved a 100% cure rate and a complete pathological and clinical response. Furthermore, PDT has proven effective against advanced stages of cancer, with 52 patients with T2N0 and T3N0 squamous cell carcinoma achieving a complete pathological and clinical response with a single PDT treatment, providing a 100% cure rate after 3 years [66].

When comparing PDT with classical chemotherapy, which is a more common choice in oncology, a study was conducted with the aim of comparing these two therapies. As a result, after comparing Photofrin-PDT with chemotherapy (5-FU and cisplatin) in the treatment of nasopharyngeal cancer, it was found that PDT demonstrated a better response in clinical criteria (*p* = 0.001). Furthermore, an improvement was also observed in the case of the Karnofsky puncture [67].

PDT shows a similar efficacy to conventional methods in treating cancers of the oral cavity, throat, nasopharynx, and larynx, as well as in cases of vascular anomalies in the head and neck region. PDT, due to the precise targeting of light on diseased areas, minimizes damage to healthy tissues. However, the PS currently used induces photosensitivity, which restricts patients after the procedure and also has limited effectiveness in treating deeper lesions. In the future, the development of PDT may focus on creating new PSs that reduce the period of photosensitivity and allow for the treatment of deeper lesions, improving patient comfort and expanding the scope of the therapy, particularly in the treatment of primary head and neck cancers [68].

#### 3.3.2. Skin Cancers

Skin cancers are a growing challenge in the medical field, due to the ever-increasing number of cases. Currently, 5.4 million new cases are observed annually in the United States alone. In general, skin cancers are divided into two main types: melanoma (cancers resulting from a dysfunction in melanocytes) and non-melanoma skin cancers (from cells derived from the epidermis), which account for about 95% of skin cancers. It is worth adding that melanoma is characterized by a very low five-year survival rate, oscillating around 15–20% [69].

In a 2008 article, the efficacy of MAL-PDT was compared to cryotherapy (which is the traditional treatment for this type of lesion) in treating superficial basal cell carcinoma (BCC). After 3 months, a 97% complete response was achieved for PDT and 95% for cryotherapy. At 5 years, recurrence rates were 22% for PDT and 20% for cryotherapy, respectively, demonstrating a superior effect of PDT on BCC, and PDT also proved to be more favorable in terms of cosmetic results, with a result of 89% compared to 50% for cryotherapy. However, when considering local PDT versus surgical intervention, the recurrence rate was higher with PDT for both superficial and nodular BCC. In a study of 196 patients with superficial BCC, the recurrence rate was 9.3% with PDT, compared with 0% with surgery. It is worth adding, however, that PDT was characterized by greater aesthetic values after the treatment, which may be of great importance for the psychological comfort of some patients [70,71].

Polymer-based nanomedicine plays a key role in modern skin cancer therapy, offering an alternative to traditional methods, especially for patients who cannot undergo surgery or intensive therapies. Polymer nanocarriers enable a more effective drug delivery to target sites, crossing the skin barrier and improving penetration into cancer cells while minimizing side effects. The development of intelligent delivery systems that use specific features of the tumor microenvironment can significantly improve the efficacy of the therapy, as well as support cancer diagnosis and prevention. In the future, advanced nanostructured systems are expected to combine multifunctional features to create personalized oncological therapies [69]. Additionally, work is underway to patent heat-assisted photodynamic therapy (HEPT) for the treatment of various skin diseases.

#### 3.3.3. Bladder Tumors

In 2020 alone, 573,278 cases of bladder cancer were diagnosed worldwide. According to the World Health Organization, this number could double by 2040. The early detection of the disease, before it spreads to the muscles, usually allows for its cure and effective control, with minimal impact on patient survival. On the other hand, muscle-invasive cancer can lead to metastases, which most often involve the lymph nodes, bones, lungs, and liver. The average survival time in such cases is about 15 months [72]. One clinical trial used hematoporphyrin-based PDT (HPD) to treat the entire bladder wall in 34 patients with refractory bladder CIS, achieving a complete response rate of 73.5% after 3 months. However, after 2 years, 77.8% of these patients had relapsed. The PDT treatment of superficial bladder cancer was generally well-tolerated, with the most common adverse events being dysuria, hematuria, and photosensitivity. In addition, some patients experienced bladder wall fibrosis or a reduced bladder capacity, which significantly reduced their quality of life [73]. In an effort to improve this approach, attempts were made to use another PS; studies of local (intravesical) ALA use show that patients with refractory bladder cancer can achieve durable complete response rates of 52–60% after 2–3 years, without the long-term photosensitivity often seen with the systemic use of other PSs [74].

PDT has the potential to treat advanced stages of bladder cancer, but non-muscle-invasive bladder cancer (NMIBC) is a particularly promising target for this therapy. The efficacy of PDT in bladder cancer, based on the experience with Photofrin-PDT, requires improvement, especially in terms of minimizing side effects. In order to optimize PDT, many variables have been considered, such as the photosensitizer, route of administration, and light conditions, to precisely target tumors in bladder tissue. However, it is important to consider the limited residence time of PSs in the bladder and the differences in bladder wall thickness between animal and human models. Despite the challenges, there is optimism for the future development of an effective PDT for NMIBC, with a holistic approach that incorporates clinical experience, advanced optics, and an understanding of the pathophysiology of the disease [75].

#### 3.3.4. Tumors in the Digestive Tract

Gastrointestinal cancers (GICs) are malignant tumors that develop in the gastrointestinal tract and digestive organs, such as the esophagus, stomach, ampulla of Vater, bile ducts, colon, and others. They account for about 30% of cancer cases worldwide. PDT is sometimes used to treat GICs because of its ability to selectively attack cancerous tissue while minimizing damage to adjacent healthy tissue, reducing systemic side effects, and allowing for multiple treatments. In a 2004 study, 102 patients with Barrett’s esophagus who had high-grade dysplasia (69 patients) or mucosal adenocarcinoma (33 patients) were treated with PDT using Photofrin^®^ as an alternative to esophagectomy. The median follow-up was 1.6 years. In 56% of patients, the complete ablation of the glandular epithelium was achieved after one full course of PDT. Esophageal strictures requiring dilation occurred in 20 patients (20%) and were the most common serious side effect. In four patients, PDT failed to ablate dysplasia or cancer, but the subsequent esophagectomy was curative in three cases. The researchers concluded that PDT is a very effective, safe, and minimally invasive first-line treatment for patients with Barrett’s dysplasia and mucosal adenocarcinoma [76].

As a result of technological advances, PDT has proven to be a safe and effective solution for the treatment of infiltrating early gastric cancer (EGC). A retrospective study from 2016 conducted in 18 Japanese centers showed that 73.7% of patients with EGC achieved complete remission after PDT. In addition, seven patients with early esophageal and gastric cancer were disease-free for 15 years after PDT treatment [77].

A prospective study of adult patients with locally advanced pancreatic cancer (LAPC) evaluated the efficacy of verteporfin-based PDT before endoscopic surgery (EUS). Patients were included if they had no significant metastases, and their tumors occupied less than 50% of the circumference of the duodenum or main artery. Of the eight patients (mean age 65 years), five had tumor necrosis 2 days after treatment, whereas three had no necrosis. Both the procedure and the follow-up period (days 1–3) were uneventful, and the patient-reported outcomes remained unchanged. The results suggest that verteporfin-based PDT may be a promising, minimally invasive treatment option for selected patients with LAPC. Recruitment and further data collection are ongoing [78].

PDT applied to the gastrointestinal tract has potential, but its development is hampered by difficulties in translating basic research into clinical practice, the complexity of dosimetry, and the lack of a large commercial sponsor. For PDT to be successful, clinical trials supported by solid sponsors, the development of cheaper and more rapidly eliminated PSs, and the use of PDT in areas difficult to access by traditional methods are necessary. Future developments may include the use of molecularly targeted agents or nanoparticles, which will increase the specificity and efficacy of the therapy [79].

#### 3.3.5. Lung Tumors

Lung cancer is one of the most commonly diagnosed cancers worldwide, with high rates of morbidity and mortality. Depending on the histology of the tumor cells, lung cancer is mainly divided into two types: small-cell lung cancer (SCLC) and non-small-cell lung cancer (NSCLC). NSCLC is the predominant type, accounting for 85–90% of all lung cancer cases, and includes various histological subtypes such as lung adenocarcinoma (LUAD), squamous cell carcinoma, and large-cell carcinoma [80].

Advanced lung cancer that is not responding to treatment can be palliatively treated with PDT in combination with chemotherapy to relieve the narrowing in the central and peripheral bronchi and to remove blockages in the lobar or segmental bronchi. The study that demonstrates this involved 12 patients (8 men and 4 women) with 13 advanced cases of non-small-cell lung cancer who were not eligible for radical surgery. They were treated with PDT in combination with chemotherapy to control local bronchial lesions. The mean age of the patients was 73.3, and the stages of the cancer varied. The median stenosis before treatment, 1 week after treatment, and 1 month later was 60% (ranging from 30% to 100%), 15% (ranging from 15% to 99%), and 15% (ranging from 15% to 60%), respectively. The median survival time was 9.3 months, and the 1-year survival rate was 30%. There were no complications or deaths related to PDT, which can be considered a great success. In a study conducted at a single institution, all patients noted an improvement in the symptoms and quality of life within a week of treatment, and there was also significant improvement in bronchial dilation and the prevention of obstructive pneumonia. PDT in combination with chemotherapy has, therefore, proven to be an effective and safe method of treating bronchial obstruction [81].

Despite the advances in PDT for the treatment of lung cancer, there are still limitations, such as precise drug targeting and light delivery methods. Future developments in PDT may include the use of PSs bound to nanoparticles, which would increase their penetration and specificity for cancer cells. Nanoplatforms equipped with receptor-based detectors, such as monoclonal antibodies, can precisely deliver PSs to lung cancer cells, which increases the efficacy of the therapy and reduces side effects. Nanomedicine supporting PDT shows promise in eliminating drug-resistant cancer cells, but further studies, especially in vivo ones, are needed to confirm its effectiveness and introduce it into clinical trials. The potential benefits of nanotechnology in PDT may lead to more effective therapy and the eradication of drug-resistant tumors, which is crucial for the future development of lung cancer treatment [82].

#### 3.3.6. Brain Tumors

Brain cancer is a serious health threat, accounting for approximately 1% of annual cancer cases in the United States. Rapidly growing and malignant brain tumors, as well as their invasion of neighboring structures, can severely disrupt critical brain functions. Available treatments, such as surgery, radiotherapy, and chemotherapy, vary in effectiveness depending on the type, location, and stage of the tumor. However, the effectiveness of these therapies is limited by challenges such as the difficulty of reaching and removing tumors from critical brain areas without damaging healthy tissue and the presence of the blood–brain barrier (BBB), which makes treatment difficult [83].

A very promising study was published in 2013, where the aim of the study was to assess the efficacy and safety of intraoperative PDT with sodium talaporfin and a 664 nm laser in patients with primary malignant brain tumors. In 27 patients, sodium talaporfin was administered before tumor resection, and then the residual lesion was irradiated. In the group of 22 patients, the 12-month survival rate was 95.5%, and the 6-month progression-free survival rate was 91%. In newly diagnosed glioblastoma multiforme (GBM), these rates were 100%. Side effects on the skin were mild and resolved within 15 days. PDT with sodium talaporfin is a promising option for supporting the treatment of primary brain tumors, especially in the case of GBM, which may be a milestone in terms of modern approaches to the treatment of tumors located in the brain [84].

The constant need to improve brain tumor treatment methods has resulted in the use of new tools such as anti-cancer vaccines, monoclonal antibodies, gene therapies, and modified CAR-T lymphocytes—a dynamically developing branch of medicine that arouses great interest among scientists and has the potential to revolutionize future methods of brain tumor treatment [85].

#### 3.3.7. Prostate Tumors

Prostate cancer affects middle-aged men, usually between the ages of 45 and 60, and is one of the leading causes of cancer death in Western countries. The diagnosis of this cancer in many men is based on a prostate biopsy, prostate-specific antigen (PSA) testing, a digital rectal examination, magnetic resonance imaging (MRI), or screening tests. Risk factors include familial predisposition, age, ethnicity, obesity, and other environmental factors. Prostate cancer is a heterogeneous disease with respect to epidemiology and genetics. Interactions between genetics, environmental, and social factors contribute to racial differences in prostate cancer survival, leading to the varying epidemiology of the disease in different regions of the world [86].

The first use of PDT in treating prostate cancer was in 1990, when tissue PSs were used. Two patients were treated: one received hemoporfin and the other sodium porfimer. After an initial resection of the prostate, PDT was performed, and, three months later, a biopsy showed no evidence of cancer. Subsequent studies of PDT for prostate cancer using different photosensitizers showed promising results, but the development of the method was hampered by financial problems of the companies producing these substances.

Temoporfin, a tissue PS, was the first to be used in a formal clinical trial of PDT in 2002. Studies have shown that PDT can cause tumor necrosis but is associated with the risk of complications such as a rectourethral fistula. ALA, which induces protoporphyrin IX, has also been investigated as a PS in PDT for prostate cancer. Initial studies have shown that ALA selectively accumulates in prostate cancer cells, and PDT leads to a decrease in prostate-specific antigen (PSA) levels [87].

PDT research in prostate cancer treatment focuses on improving the targeting PS to tumor cells and minimizing the toxicity in healthy tissues. Passive targeting uses nanoparticles such as liposomes and inorganic nanomaterials that improve the permeability and retention (EPR) effect in tumors. However, the efficacy of EPR is controversial, and the active targeting of specific tumor cell receptors such as Prostate-Specific Membrane Antigen (PSMA), integrin αvβ3, or Cation-Independent Mannose-6-Phosphate Receptor (CI-M6PR) is more precise and effective. Studies on new PS ligands and conjugates, as well as gold nanoparticles or iridium complexes, show promising results in increasing the efficacy of PDT. However, challenges related to the PS accumulation in tumors and side effects require further investigation to make PDT an effective treatment option [88].

#### 3.3.8. Dental Treatment

Peri-implantitis is an inflammatory condition of the tissues around oral implants that can lead to bleeding, damage to the alveolar bone, and the loosening of the implants, posing a serious risk to the success of the implantation. The causes of peri-implantitis are related to the presence of bacterial plaque, and patients with periodontal disease may exhibit the same pathogens around the implants. The key to treating this condition is the effective removal of plaque pathogens. Current methods include mechanical therapy, antibiotic therapy, laser therapy, and aPDT.

**Figure 5:**
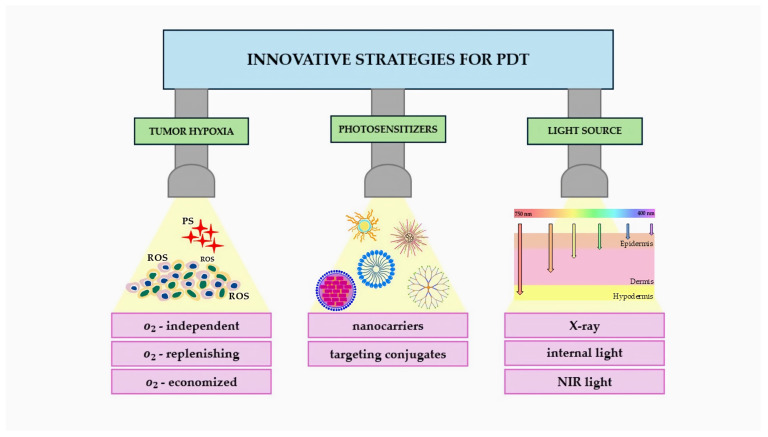
Innovative strategies for PDT.

Numerous studies have shown that aPDT is effective in treating peri-implantitis, improving periodontal indices and accelerating regeneration compared to traditional methods. PDT significantly reduces pathogenic periodontal bacteria, such as *Actinobacillus actinomycetemcomitans* and *Porphyromonas gingivalis*, and reduces inflammation, bleeding, and the probing depth. This therapy has also been shown to be an effective complement to surgical treatment, offering better results compared to mechanical debridement [89]. There is a need for the continuous improvement of this method, through increased clinical trials, in order to gather as much information as possible on key parameters that can improve and establish the best treatment option for peri-implantitis using PDT (Figure 5).

### 3.4. Photosensitizers

**Figure 6:**
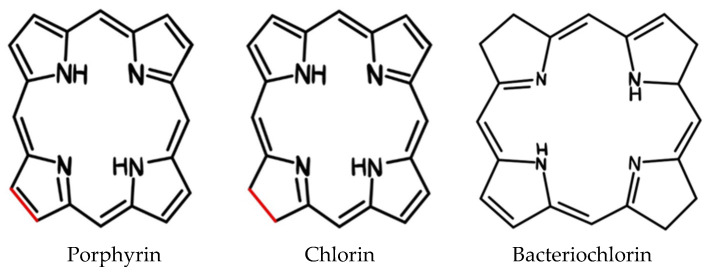
Structural formula of porphyrin, chlorin, and bacteriochlorin.

The photosensitizer is introduced into the body as a substance that appears to have no effect. The main goal is to lead or induce pathological cells into a programmed death pathway [90,91]. After a certain period of time, the PS reaches its highest concentration in the area with cancer compared to the surrounding healthy tissue. Then, the area containing tumor tissue with a photosensitizer is irradiated with light with a wavelength that corresponds to the maximum of the PS absorption spectrum in the spectral range above 500 nm. The choice of light source must be adjusted like this so that its emission band coincides with the absorption band of the dye, which is necessary in order to induce a photochemical reaction (Table 1). As a result of photon absorption, the PS molecule changes from the basic energy state to the singlet excited state. In the context of PDT, there are two important routes to deactivate a molecule from this state to the ground state: A PS molecule in an excited singlet state can return to the ground state, releasing excess energy in a radiative process, which is called fluorescence. Detecting the fluorescence of a drug accumulated in tumor tissue allows for precise diagnostics, enabling the determination of the shape, size and location of the cancer lesion (Figure 6).

As a result of the transition to the triplet state, the photosensitizer molecule excited to a singlet state is converted to a triplet state. The lifetime of a PS molecule in the triplet state is long (hundreds of microseconds) and interacts with oxygen. In the ground state, the oxygen molecule is in the triplet state and effectively deactivates the triplet state of the drug, generating a strong oxidant—oxygen singlet (Table 2). In this way, the process of deactivation of the PS molecule can lead to the generation of singlet oxide, which is a powerful oxidant, particularly effective in the destruction of cancer cells in PDT [92].

For a PS to be effectively used in the diagnosis or treatment of cancer, it must meet several key conditions:

1. Selective accumulation in tumor tissue: The PS should be able to selectively accumulate in the area of tumor tissue, minimizing the effect on healthy tissues;
2. No phototoxic effects in healthy tissues: The PS should not cause undesirable phototoxic effects in healthy tissues, which means that it cannot damage healthy cells when exposed to light;
3. Appropriate absorption bands: The absorption bands of a PS should not coincide with the absorption bands of the body’s natural pigments, such as melanin or hemoglobin, or with the absorption bands of water in the area close to infrared;
4. Efficient generation of singlet oxygen and oxidative reactions: The PS should be able to efficiently generate singlet oxygen and other oxidative reactions that are crucial in the destruction of cancer cells;
5. Minimal side effects: The PS should not cause significant side effects that may be harmful to the patient;
6. Most PS also accumulate in many host organs, e.g., the liver. Since these sites are not usually irradiated, no damage occurs. The PS should be low in toxicity and easily removed from the body after the completion of therapy to minimize the side effects and burden on the patient’s body.

In the 1970s, hematoporphyrin (Hp) and mixtures of hematoporphyrin derivatives (HpD) were the most commonly used PSs, known as the first generation of PSs [93].

**Figure 7:**
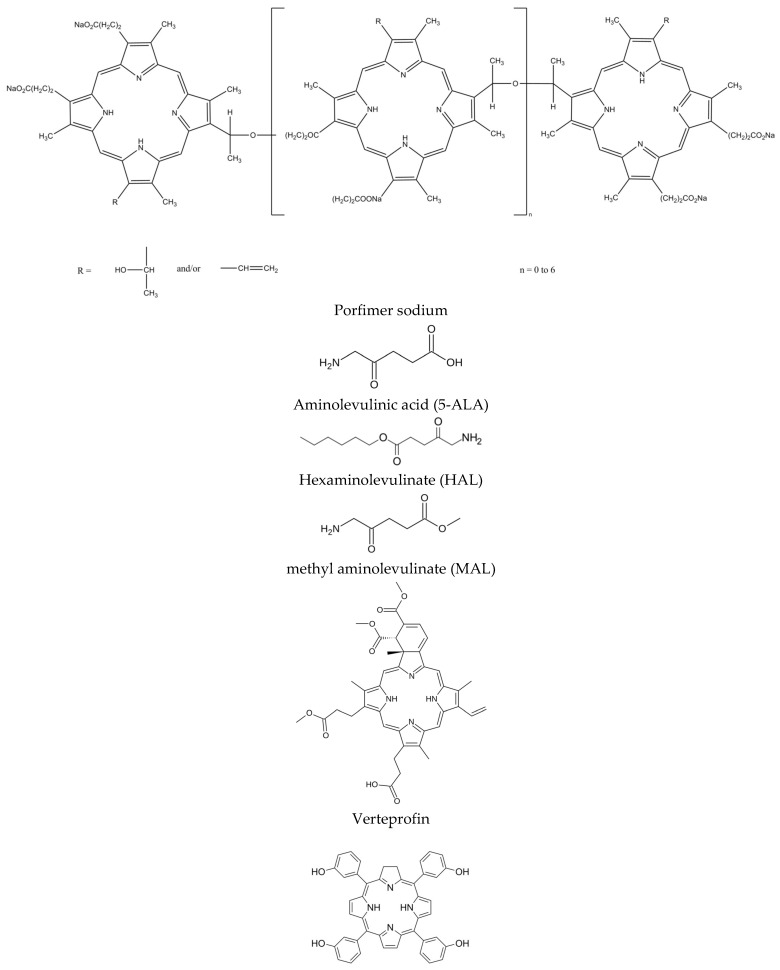

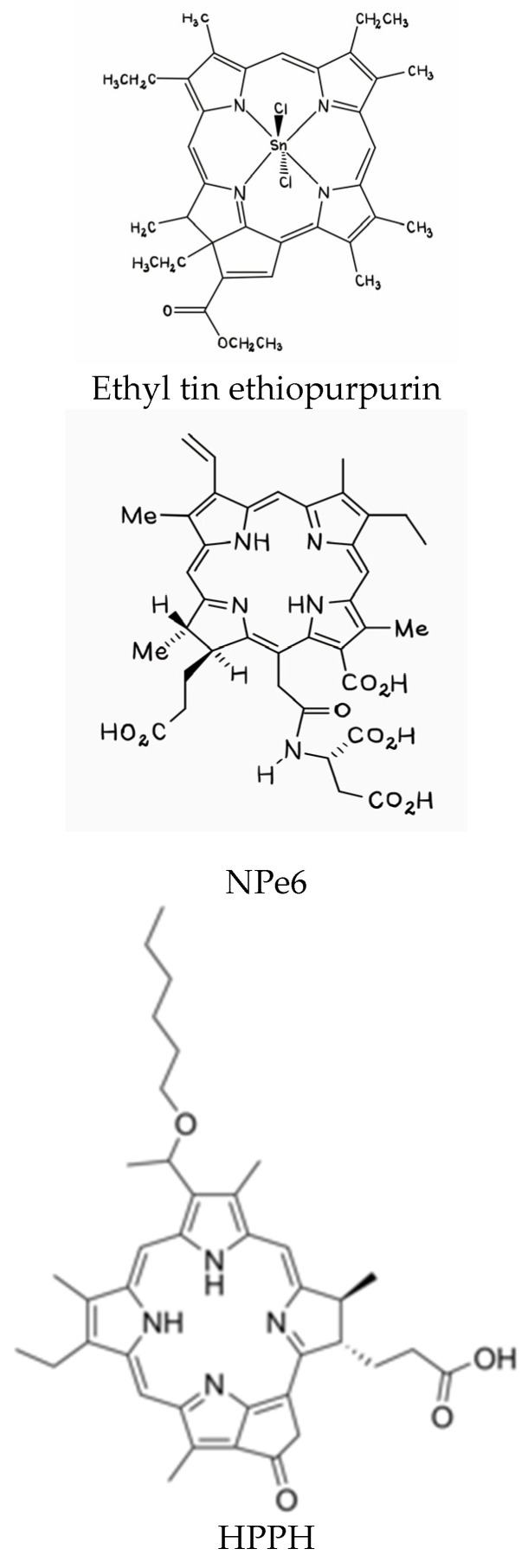
The chemical structure of clinically available photosensitizers.

In the 1980s, the second generation of photosensitizers from various chemical families was created, such as TPPSn-sulfonated tetraphenylporphyrin [94], zinc and aluminum phthalocyanines [95], m-THPC-meso-tetra(hydroxyphenyl) chlorins [96], chlorin e6 [97], m-THPP—meso-tetra(3-hydroxyphenyl) porphyrin [98], merocyanine [99], hypericin [100], or methylene blue derivatives [101]. Chlorins, as one of the chemical groups within porphyrin compounds, are formed by reducing the double bond in one of the four pyrrole rings. This modification causes chlorins to absorb light much more intensively in the long-term spectral range, which is of key importance in anti-cancer PDT. Chlorins can be stimulated with longer-wavelength light than porphyrins, which allows the deeper penetration of light into tissues compared to porphyrin PS [102]. Chlorins reach their maximum concentration in the tumor area after a few hours, such as chlorin e6, which reaches its maximum after about 3 h and is then relatively quickly eliminated from the body within 24–48 h [103]. The chemical structure of clinically available photosensitizers is presented in Figure 7.

**Table 1:** Examples of in vivo and in vitro experiments on porphyrins and porphyrin derivatives as photosensitizing agents in photodynamic therapy.

Photosensitizer	Nanoparticle	Results	In Vivo/In Vitro
Photofrin^®^	F3—Polymer-targeted particles (F3: 31-amino acid vascular homing peptide targeting nucleolin on tumor vasculature)	High rate of uptake of nanoparticles by cells Significant improvement in survival rate (MDA-MB-435 cell line—breast cancer, 9L rat gliomas)	In vitro [104]
	Nanoporous zinc oxide	Increased ROS generation Increased cytotoxic effect (Cell line A549—lung cancer)	In vitro [105] In vivo
	Liposomes	Higher phototoxic effect of liposomal photofrin compared to the free drug (Athymic nude rats, Cr:NIH-rna strain with U97 cells)	In vivo [106]
Protoporphyrin IX	Gold particles	Increased cytotoxic effect of conjugates (HeLa cell line—cervical cancer) Increased apoptosis (HeLa cell line—cervical cancer) Increased single oxygen generation (male Newborn Medical Research Institute [NMRI] mice)	In vitro [107] In vitro [108] In vivo [109]
	Polyethyleneimine nanoparticles	Ability to generate single oxygen upon exposure to light with a wavelength of 635 nm	In vitro [110]
	Carbon particles	Increased single oxygen generation Additional bioluminescence effect Increased phototoxic effect (MMC-7721 cell line—hepatocellular carcinoma)	In vitro [111]
	Nanoparticles with a silver core and a silica coating	Increased single oxygen generation (U251MG cell line—astrocyma glioblastoma, HepG2 cell line—hepatocellular carcinoma)	In vitro [112]
	Polymerosomes	Increased cytotoxic effect Selective cytotoxic effect on melanoma cells (Cell line A375—malignant melanoma)	In vitro [113]
	Micelle of poly(ethylene glycol)-polycaprolactone (PEG-PCL)	Synergistic activity with erlotinib (MDA-MB-231 cell line—breast cancer)	In vitro [114]

**Table 2:** In vivo and in vitro experiments on chlorins and chlorin derivatives as photosensitizing agents in photodynamic therapy.

Photosensitizer	Nanoparticle	Results	In Vivo/In Vitro
Chlorin e6	Lipidots	Reduced dark toxicity Retained phototoxicity (CAL-33 cell line—squamous cell carcinoma of the tongue)	In vitro [115]
	Superparamagnetic iron oxide partition nanoclusters (SPION)	High solubility in water Single oxygen generation preserved Significant delay in tumor growth (4T1 cell line—breast tumor mice, female nude mice carrying 4T1)	In vitro [116] In vivo
	Methoxy-poly(ethylene glycol)-poly(D,L-lactide) (mPEG-PLA-Ce6)	Increased single oxygen generation Increased cellular internalization (A549 cell line—lung cancer, monolayers and 3D spheres)	In vitro [117]
Verteporfin	Poly(D,L-lactide-co-glycolide)	Size-dependent toxicity Increased phototoxic effect for smaller nanopartitions Efficiently controlled tumor growth by small nanopartitions loaded with verteporfin (EMT-6 cell line—mammary tumor mice, SKH1 female nude mice)	In vitro [118] In vivo
2-(1-Hexyloxyethyl)-2-devinyl pyropheophorbide-a (HPPH)	Functionalized polyacrylamide (AFPAA)	Efficient encapsulation, post-loading, or HPPH conjugation Highest phototoxicity and single oxygen production for the post-loaded form No dark toxicity observed Tumor location in a murine colorectal cancer model (PC-3 cell line—prostate cancer, MDA-MB-435S cell line—melanoma, mice carrying human glioblastoma U87MG)	In vitro [119] In vivo

An interesting case of a substance used in PDT is acid 5-ALA. It has been approved for therapeutic use by the US FDA in 1999. It is used in the treatment of skin cancer and is applied topically, directly to the tumor area in the form of an ointment. After a few hours in the skin, as a result of natural processes, protoporphyrin is generated, which acts as a photosensitizer. Importantly, ALA is a precursor of hematoporphyrin synthesis in the body, and the resulting porphyrin is not toxic to the body in the absence of light.

As a result of numerous scientific studies, it has been proven that the photosensitizers with amphiphilic characteristics show the most favorable properties. These substances have two important features in their structure: lipophilic domains and side domains chains that exhibit hydrophilic properties (Table 3). The combination of these two different properties makes amphiphilic photosensitizers perfectly adapted to accumulate in cancer cells. One part of the PS molecule interacts with the lipid structures of the cell, while its side groups are located in the aqueous part of the cell. As a result of this, they can accumulate effectively in cancer cells, which is crucial in the context of photodynamic therapy.

### 3.5. Photosensitizers—New Trends

In recent years, there has been a significant increase in interest in the use of nanoparticles in PDT due to their unique physicochemical properties that can significantly improve the efficacy of cancer treatment. In particular, nanoparticles such as gold nanoparticles, titanium dioxide nanoparticles, and quantum dots are increasingly used as carriers for photosensitizers. Due to their plasmonic and semiconducting properties, these nanoparticles can increase light absorption, which, in turn, enhances the production of ROS in cancer tissues. Moreover, due to their small size and ability to be functionalized, these nanoparticles exhibit an improved distribution profile in the body, which allows for the more precise delivery of PSs to cancer cells while minimizing the side effects on healthy tissues. A 2022 review of the treatment of human squamous cell carcinoma with nanoparticle PDT showed that this type of treatment can be easily combined with other treatments for this cancer. In this regard, nanoparticle-conjugated PSs have been proven to be effective in both in vitro and in vivo animal studies in human SCC, similar to the PDT used in combination with a nanotechnology-based drug delivery system. Furthermore, the combination of therapies has been shown to be more effective than a single therapy alone [134]. In a study conducted in 2023, the disinfection efficacy and bond integrity in caries-affected dentin (CAD) were assessed using different methods, including methylene blue (MB), photodynamic therapy (MB-PDT), carbon nanoparticles combined with MB (MB@CP-PDT), and Er, Cr: YSGG laser (ECL). CAD samples were subjected to different disinfection methods, followed by shear strength tests (SBS), morphological analysis (SEM), X-ray spectroscopy (EDX), and damage analysis. The results showed that the use of MB@CP-PDT resulted in the highest reduction in the level of Lactobacillus bacteria, which confirms the high antibacterial efficacy of this method and makes it a promising solution in the context of disinfection in dentistry [135].

Liposomes and nanolipids are another innovative trend in the design of PS delivery systems in PDT. Liposomes, which are spherical structures composed of lipids, and nanolipids, i.e., smaller lipid forms, are used to encapsulate PSs, which provide their protection against premature degradation in the body and allow for a more controlled release of the active substance at the site of action. Such nanocarriers are particularly valuable in the context of cancer therapy, because they can increase therapeutic efficacy by improving the stability of photosensitizers in the bloodstream and their selective delivery to cancer tissues. Additionally, liposomes and nanolipids can be modified to increase the specificity of targeting specific types of cancer cells, which additionally enhances the effectiveness of the therapy [136]. A 2010 study evaluated the effect of PDT on collagen network organization and inflammatory infiltration in advanced chronic periodontal disease using two different drug delivery systems: liposomes and nanoemulsions. In the group of patients treated with liposomes (group L), a significant increase in the fraction of collagen fiber area in the gingival tissue was noted, amounting to 66 ± 19%, compared to the control group (35 ± 21%, *p* < 0.02). In addition, the number of macrophages in this group was significantly reduced (*p* < 0.05) compared to the control group. In the group of patients treated with nanoemulsions (group N), the fraction of collagen fiber area was 56 ± 23%, which did not differ significantly from the value observed in the control group (44 ± 23%). In group N, a significant reduction in the number of Langerhans cells was also observed (*p* < 0.02). These results indicate that the use of liposomes as a carrier of PSs in PDT leads to a more significant reorganization of the collagen network and modification of the inflammatory response compared to nanoemulsions.

One area of intensive research is multifunctional PSs, which are an innovative approach combining therapeutic and diagnostic functions, also known as theranostics. This is an approach that integrates therapy with diagnostics, allowing for the simultaneous treatment and monitoring of the effects of therapy. Theranostic PSs are designed so that when activated by light they can induce a therapeutic effect, for example, by generating reactive oxygen species that destroy cancer cells. At the same time, the same compounds can be used for diagnostics, emitting signals that can be tracked using medical imaging techniques such as magnetic resonance imaging (MRI) or computed tomography (CT). This allows for the monitoring of the effects of therapy in real time, which allows for the tailoring of the treatment to the individual needs of the patient. In a study conducted in 2017, standard PDT (662 nm) was compared with a new approach in which the fluorescence of the PS chlorin E6 accumulation in tumor tissue (408 nm) was controlled. In the study group (SG), 96% of patients showed intense fluorescence, and the total exposure time was significantly shorter (365 ± 65 s) than in the control group (690 ± 65 s). The differences in treatment outcomes between groups were statistically insignificant. These strategies suggest that fluorescence control can optimize and individualize PDT [137].

Another promising direction in photosensitizer research is compounds that respond to specific conditions in the tumor microenvironment. Cancers have a unique microenvironment that differs from healthy tissues, for example, in terms of pH, oxygen availability, and the presence of specific enzymes. Environmentally responsive PSs can be designed to activate only in the presence of these specific conditions, which increases the selectivity of the therapy and minimizes the damage to healthy tissue. An example is PSs that become active only in the acidic environment characteristic of many types of cancer. Another example is compounds that are activated by specific enzymes present in cancer cells, which allows for the precise targeting of the therapy.

**Figure 8:**
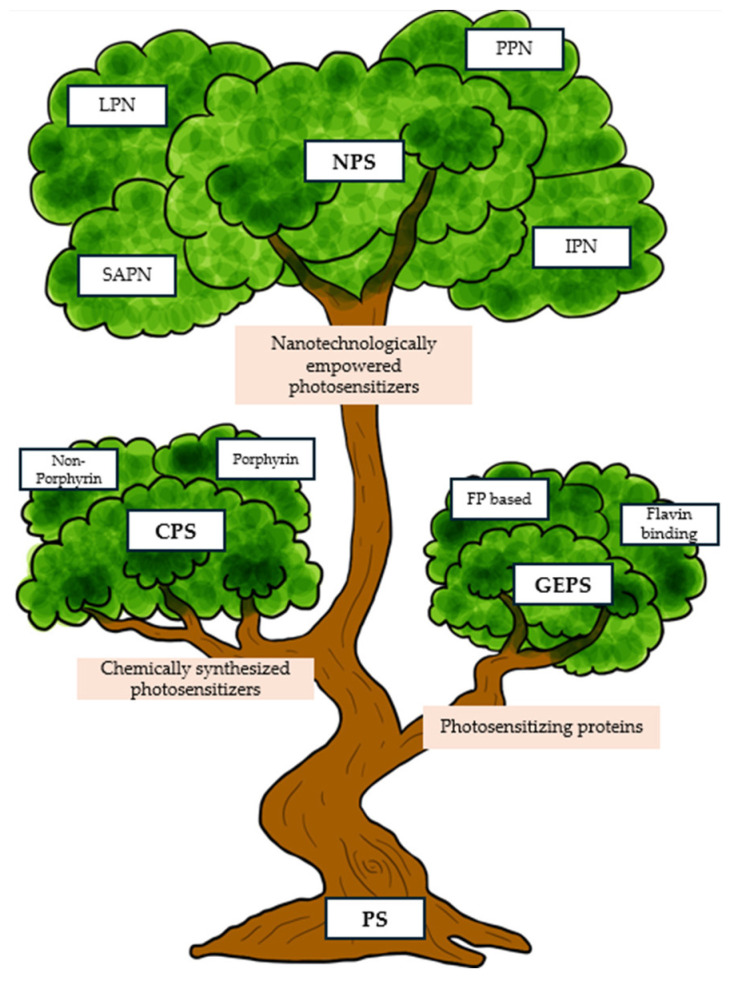
Photosensitizers for clinical and non-clinical applications. NPS: nanotechnologically empowered photosensitizers; PPN: polymeric nanoparticles; LPN: lipid-based nanoparticles; SAPN: self-assembling peptide nanoparticles; IPN: inorganic-based nanoparticles; CPS: chemically synthesized photosensitizers; GEPS: genetically engineered photosensitizers; FP-based: fluorescent-protein-based photosensitizers.

The new-generation PSs represent a significant step forward in the field of PDT, bringing a number of innovative solutions that significantly improve therapeutic efficacy and selectivity (Figure 8). One of the key directions of development is PSs based on organic dyes. New dyes, especially those capable of absorbing light in the near-infrared (NIR) range, are gaining importance due to the possibility of deeper light penetration in tissues. This is crucial because it allows for the more effective treatment of cancers located deep in the body, which were previously difficult to achieve using traditional methods. Additionally, these dyes are characterized by better photophysical properties, such as the higher quantum efficiency of singlet oxygen generation, which translates into a greater therapy efficacy. One example is BODIPY. This is a class of organic dyes that, due to their unique properties, such as strong fluorescence and exceptional photochemical stability, attract attention in research on new methods of cancer treatment. Due to the possibility of structural modifications, BODIPY allows for the tuning of the absorption and emission wavelengths, which enables the design of molecules with specific properties, ideal for various medical applications [138].

In parallel, chemically modified versions of classical PSs, such as porphyrins and chlorins, are being developed. These modifications lead to a significant increase in selectivity for cancer cells, minimizing the risk of damage to healthy tissue. In addition, chemical modifications improve the photodynamic properties of these compounds, which include the increased production of ROS, better chemical stability, and a more controlled release of active oxygen in response to specific conditions of the tumor microenvironment, such as low pH. These improved properties make these PS more effective and precise in therapy, opening up new possibilities in the treatment of cancers and other diseases that have been difficult to effectively treat so far. In 2010, a study was conducted on silicon phthalocyanine Pc 4 (Pc 4). It was evaluated for its efficacy and safety in PDT of skin cancers. The study included 43 patients with various skin cancers, including actinic keratosis, Bowen’s disease, squamous cell carcinoma, basal cell carcinoma, and mycosis fungoides. Each patient received a single treatment with Pc 4-PDT, and the effects of therapy were monitored for 14 days. The results showed that Pc 4-PDT was well-tolerated, without significant local toxicity or increased photosensitivity. Particularly significant results were observed in the treatment of mycosis fungoides, where 14 of 35 patients showed a positive clinical response, associated with the induction of tumor cell apoptosis, measured by increased caspase-3 activity [139].

PSs targeting specific receptors are an advanced strategy in photodynamic cancer therapy, the aim of which is to increase the selectivity and therapeutic efficacy. As part of this strategy, immunophotosensitizers are being developed, which are created by conjugating PSs with antibodies or ligands specific for receptors present on the surface of cancer cells. This approach allows for the precise targeting of PSs to cancer cells, which minimizes the damage to healthy tissues and increases the efficacy of therapy. Additionally, designing PSs targeting mitochondria allows for their specific accumulation in these organelles, which leads to the more effective induction of apoptosis in cancer cells. By destabilizing mitochondrial functions and initiating apoptotic pathways, these PSs contribute to the increased efficiency of cancer cell elimination while limiting the side effects in healthy tissues. Both of these strategies for targeting PSs aim to optimize photodynamic cancer therapy, which may lead to improved clinical outcomes and a reduced risk of side effects. In 2013, a study was conducted using Radachlorin^®^ in patients with advanced non-small-cell lung cancer (NSCLC) and central airway obstruction. Radachlorin^®^, a second-generation PS with faster pharmacokinetics, was administered at a dose of 1 mg/kg, 4 h before laser irradiation at 200 J/cm^2^ for 11 min. The results showed improvement in airway obstruction and an increase in FEV1 in most patients, with no major complications. The 1-year survival rate was 70%, confirming the efficacy of Radachlorin^®^ in the palliative treatment of NSCLC [140].

Therapies targeting cancer mitochondria, especially in the context of PDT, have shown greater efficacy than non-targeted methods. Mitochondrial photosensitizers are key because they help overcome hypoxia, which improves the efficacy of the treatment [141]. An example is indocyanine dyes, such as IR-780 derivatives, which absorb far-red light well, making them suitable for PDT. The problem is their tendency to aggregate in water, which can reduce stability and efficacy. A solution is to encapsulate these dyes in nanocarriers, such as hyaluronic acid (HA), which is effective in targeted drug delivery to cancer cells overexpressing CD44 receptors [142]. A new indocyanine derivative, IR-Pyr, has been developed that preferentially accumulates in mitochondria and shows improved photostability. The use of the IR-Pyr complex with HA enables the precise delivery to tumors, which was confirmed in in vitro and in vivo studies, increasing the efficacy of PDT therapy [143].

Two-step activated photosensitizers are an innovative approach in PDT, enabling precise control over the therapeutic activation process. One of the solutions being developed in this field are photosensitizers that require activation by two different wavelengths of light. This type of approach allows for the more precise control of the time and place of activation, which consequently minimizes the risk of damage to healthy tissues. An example of such a mechanism is the use of one wavelength to selectively activate the photosensitizer in a specific location, and then the use of the second wavelength to trigger the appropriate therapeutic process.

Another advanced strategy is PSs whose activation depends on a combination of light and a specific chemical stimulus. In this case, the PS requires both irradiation and the presence of a specific chemical agent to initiate the reaction. This approach allows for the even more precise targeting of the therapy, because activation occurs only in places where both conditions are met—the availability of light of the appropriate wavelength and the presence of a given chemical agent. An example would be a PS which requires both the exposure to light and the presence of heavy metal ions characteristic of diseased cells, which provides an additional selectivity of action.

Long-acting PSs are chemical compounds characterized by an extended half-life in the body, which allows for longer therapeutic activity after their administration. The increased stability and extended duration of action of these compounds allow for the maintenance of the therapeutic effect for a longer period, which is particularly important in the context of photodynamic cancer therapy. This reduces the frequency of necessary therapeutic interventions, which can lead to increased patient comfort and improved treatment efficiency. The prolonged action of PSs allows for the more precise planning of therapeutic regimens, allowing for the optimal adjustment of doses and the schedule of administration, which, in turn, minimizes the risk of adverse effects. Consequently, the use of long-acting PSs is a significant step forward in the field of therapy, offering more effective and less burdensome treatment methods for the patient.

In 2001, a team led by Tang discovered the phenomenon of aggregation-induced luminescence (AIE), which is where some fluorescent molecules exhibit weak light emission in the dispersed state, but their fluorescence increases upon aggregation [144]. This mechanism is based on the fact that, in the dispersed state, the excited energy of the molecules is dissipated by their rotation and vibration, resulting in weak fluorescence emission [145]. However, in the aggregated state, intramolecular motions are restricted, which prevents energy dissipation and leads to increased light emission. This discovery enabled the development of numerous AIEgens, such as tetraphenylethylene (TPE) [146], thiophene (TP) [147], distyryl anthracene (DSA) [148], and tetraphenylpyrazine (TPP) [149], which, unlike traditional PSs, are characterized by stability and strong fluorescence in aggregated states. In recent years, three mono-hydroxycorrols (1–3) and their complexes with gallium (III) (Ga1–3) have been synthesized and studied for their photodynamic anti-cancer activity against breast cancer cells. The results indicated that all tested corrols showed high cytotoxicity against MDA-MB-231 and 4T1 cell lines after irradiation with 625 nm light. In particular, the Ga3 complex showed excellent phototoxicity and selectivity towards MDA-MB-231 cells, with an IC50 of 0.06 ± 0.03 μM and a selectivity coefficient of 1338.83 against normal human Huvec cells. The efficiency of Ga3 even exceeded the clinical drug used in PDT, m-THPC. Preliminary studies of the mechanism of action suggest that corrol 3 and Ga3 mainly localize in the cytoplasm, where, upon irradiation, they generate ROS, leading to mitochondrial membrane depolarization and cell cycle arrest in the sub-G1 phase. A flow cytometric analysis confirmed that corrol 3 and Ga3 induce the apoptosis of cancer cells after photodynamic therapy, while showing minimal cytotoxicity in the dark. These results suggest that corrol 3 and Ga3 are promising candidates for use in the PDT of breast cancer [150].

Data from 2021 indicate that strategies are being developed to increase oxygen delivery or to use PSs that act independently of oxygen. For example, the TOOKAD^®^ soluble PS [151], clinically approved in several countries, generates mainly superoxide and hydroxyl radicals instead of singlet oxide, which allows it to be effective in hypoxic conditions [152]. Similarly, TLD-1433 shows phototoxic activity even at low oxygen levels, suggesting the potential of these compounds in clinically relevant hypoxia. The further development of PSs that can function effectively in oxygen-poor environments is ongoing, which could enhance the efficacy of PDT in difficult-to-treat cancers [153]. Currently, research is underway to develop PSs devoid of heavy atoms such as ruthenium, iridium, iodine, and bromine, the presence of which may increase the risk of toxicity in the dark, reduce photostability, shorten the lifetime of the excited triplet state, and increase production costs [154].

## 4. Discussion

### 4.1. Advances in Imaging Techniques and Diagnostics Supporting PDT

The effectiveness of PDT requires accurate diagnostics and monitoring the treatment process; therefore, progress in imaging techniques and diagnostics plays a key role in supporting PDT.

- Imaging using fluorescence techniques:

Advanced fluorescence imaging techniques are now available for visualizing and monitoring the accumulation of PSs in tissues. An example is fluorescence computed tomography (FLT), which allows the three-dimensional reconstruction and localization of PSs in patients’ bodies [155].

- Optical imaging:

Optical imaging techniques such as frame spectroscopy enable the identification and assessment of tissue biomarkers [156].

- Advanced microscopy imaging:

The use of microscopy imaging allows for the analysis of tissue morphology on a microscale and the observation of cell reactions to PDT. This helps doctors tailor the therapy to the patient’s individual needs [157].

- Molecular diagnostics:

The use of molecular biology techniques, such as testing the level of gene expression or protein analysis, allows for the personalization of PDT. The appropriate PSs and the optimal time for therapy can be determined [158].

- Imaging using hybrid technologies:

A combination of different imaging techniques, such as computed tomography and magnetic resonance imaging with optical techniques, allows the precise localization of areas to be treated and monitoring changes in real time [159].

### 4.2. The Most Important Centers Specializing in the Treatment of Skin Problems with Photodynamic Therapy in the World

Table 4 below is presenting data of the most important centers specializing in skin PDT in the world.

### 4.3. Side Effects and Complications of PDT

Despite its advantages, photodynamic therapy, like many other medical procedures, may be associated with some side effects and complications. The most common include skin burns in the treated area, especially in the case of excessive exposure or photosensitivity. Patients undergoing PDT may also experience pain and discomfort during the procedure itself and after its completion; they may also be more sensitive to sunlight for some time after the procedure, which may lead to solar burns [160]. In addition, the skin may become red and swollen at the treatment site and may peel and itch. In some cases, PDT may leave permanent scars or skin discoloration [161]. There is a risk **of** infection at the site of treatment, especially if the wounds are not properly protected and nurtured [162]. Please note that the side effects of PDT may vary depending on the type of disease, area of treatment, and individual patient’s reaction. There is always a risk of more serious complications, so it is important to carry out therapy under the supervision of an experienced specialist.

### 4.4. The Future of PDT

There is now a clear and growing interest, as well as an international research effort, focused on developing new PSs, investigating the mechanisms of PDT at the molecular level, increasing its effectiveness through combined therapies, and assessing potential clinical indications [163]. Although there are now regulatory approvals for the clinical use of PDT PSs in many countries around the world and light applicators, the overall number of approved clinical indications is still limited. It is expected that the pharmaceutical industry and research institutes will continue to conduct numerous clinical trials to evaluate the applications of PDT, both as a complement to and as a replacement for traditional oncological and non-oncological treatments. Optical methods and nanotechnology will continue to play an important role in the characterization of target tissues, determining the PDT dose and assessing treatment results. Combination therapies, personalized treatment planning, and dosimetry will certainly continue to be an important element of PDT.

In 1993, Canada became the first country in the world to approve Photofrin^®^ for the treatment of bladder cancer. Approvals soon followed in the Netherlands and France, where the drug was used to treat advanced esophageal and lung cancer. Germany focused on treating early-stage lung cancer, while Japan expanded the use of Photofrin^®^ to esophageal, stomach, and cervical cancer, and cervical dysplasia. Clinical trials in Canada have shown that PDT with Photofrin^®^ significantly reduces the rate of bladder cancer recurrence after surgery, despite side effects such as photosensitivity and urinary symptoms. These results were so promising that the need for further research on dose optimization was suggested.

In 1995, the FDA approved Photofrin^®^ for the treatment of partially obstructive esophageal cancer after the successful completion of phase three clinical trials. PDT therapy demonstrated longer tumor response times and more complete responses compared to Nd-YAG laser ablation, with fewer treatments. A year later, Biel’s studies of early-stage head and neck cancer showed a complete response in patients with superficial cancer of the larynx and other sites in the head and neck. In 1997, the largest study of PDT in the treatment of superficial esophageal cancer was conducted and showed a significant reduction in dysplasia in patients with Barrett’s esophagus.

Another breakthrough was the FDA approval in 1999 of verteporfin (Visudyne^®^) for the treatment of submacular neovascularization associated with AMD. At the same time, PDT with ALA and MAL began to be used in dermatology for the treatment of actinic keratosis, achieving high effectiveness.

Between 2001 and 2005, the European Medicines Agency (EMA) approved Foscan^®^ for the treatment of advanced squamous cell carcinoma of the head and neck. Studies on PDT with verteporfin in the treatment of hemangiomas have shown significant improvement in patients’ condition. At the same time, research began on PDT for the treatment of cholangiocarcinoma (CC) and on newly developed drugs such as Tookad^®^ and its derivative WST-11, which ushered in a new era in therapy as a result of their rapid activation in blood vessels.

In 2006, studies on PDT with Photofrin^®^ in the treatment of lung cancer showed an increased expression of VEGF and MMPs, suggesting that their inhibition may increase the effectiveness of the therapy. A year later, PDT was used in dermatology and microbiology, and research on actinic cheilitis and Bowen’s disease showed the high effectiveness of PDT with MAL.

In 2009, PDT was recognized as a promising treatment for localized nasopharyngeal carcinoma (NPC), and studies in anal cancer demonstrated the effectiveness of PDT with Photofrin^®^ in its treatment, minimizing pain and complications. The years 2010–2015 brought further successes in the research on PDT with 5-ALA and mTHPC in the treatment of potentially malignant oral diseases and in the treatment of papilloma acuminata. The effectiveness of PDT in the treatment of warts and peri-implantitis has also been confirmed. In 2015, a study of PDT with MAL and imiquimod in the treatment of actinic lesions showed that both methods were effective, but patients preferred PDT with MAL.

The year 2016 brought confirmation of the safety and effectiveness of PDT with HPPH in the treatment of the early stages of laryngeal diseases, as well as a significant improvement in the condition of patients with port-wine stains as a result of PDT with hemoporfin. In 2017, PDT was shown to be highly effective in the treatment of vulvar lichen sclerosus, and, in 2018, in the treatment of rosacea. A 2019 study on combining PDT with minimally invasive spinal stabilization (VCF) procedures found that PDT can improve mechanical stability (Table 5).

The last five years of research have shown that PDT effectively treats warts and reduces the rate of recurrence. In 2022, studies on PDT in the treatment of herpetic gingivitis showed a significant improvement in the condition of patients, and, in 2023, PDT with indocyanine green (ICG) in the treatment of keloids effectively inhibited the cellular activity of keloid fibroblasts. Moreover, research on PDT in the context of SARS-CoV-2 confirmed that the therapy is safe and effectively reduces the infectivity of the virus.

PDT is a promising method for cancer treatment, but its efficacy is limited by several barriers that are currently the focus of intensive research. One of the most significant challenges is the limited penetration depth of light. PDT utilizes photosensitizers that are activated by light with wavelengths in the 600–800 nm range. In this range, light penetrates tissue to a relatively shallow depth, typically around 1 cm, which makes the treatment of deep-seated tumors challenging. Several strategies are being explored to enhance the effectiveness of PDT for deeper tumors. These include using longer-wavelength light (e.g., near-infrared), which can penetrate tissues more effectively, and advanced fiber-optic technologies that allow precise light delivery to tumors located in hard-to-reach areas. Additionally, implantable and wearable light-emitting devices, such as LEDs, are gaining increasing attention for providing the continuous and controlled illumination of tumors.

Another important limitation of PDT is the side effects associated with photosensitizers, especially prolonged photosensitivity, which can last for several weeks after therapy. To reduce the risk of these side effects, new generations of photosensitizers with shorter elimination times from the body are being developed. One example of such substances is ALA derivatives, which are metabolized faster than traditional photosensitizers, significantly reducing the time during which patients are at risk of photosensitivity. Furthermore, photosensitizers that are activated only by specific wavelengths of light are being developed, minimizing the risk of accidental activation by sunlight [164].

The nonspecific distribution of photosensitizers in the body also poses a significant problem, as it can lead to damage to healthy tissues surrounding the tumor. In response to this challenge, various methods are being developed to target photosensitizers directly to cancer cells. One such strategy involves the use of monoclonal antibodies, which can precisely deliver photosensitizers to cancer cells, thereby reducing their accumulation in healthy tissues. The application of nanotechnology is also showing promising results. Nanoparticles can serve as carriers for photosensitizers, not only improving their targeting precision but also increasing drug concentration at the tumor site. An example of such carriers includes nanoparticles containing protoporphyrin IX (PpIX), which have demonstrated significantly higher therapeutic efficacy and reduced toxicity to surrounding healthy tissues [165].

A final key challenge for PDT is tumor hypoxia, which reduces the effectiveness of the therapy. The photodynamic process requires the presence of oxygen to generate ROS that cause cancer cell destruction. Many tumors, particularly larger ones, exhibit areas of hypoxia, which significantly limits the efficacy of PDT. Several strategies have been developed to address this issue by improving the oxygen availability in the treated areas. One such approach involves the use of oxygen-releasing substances near the tumor to increase the oxygen availability during illumination. Simultaneously, work is being carried out on photosensitizers that remain effective even in low-oxygen conditions, which could be crucial for improving the efficacy of PDT in the future.

These innovative approaches significantly expand the potential of PDT and bring this treatment closer to broader clinical application. PDT is particularly promising for treating superficial cancers and in cases where other treatment methods are less effective or too invasive. The introduction of nanotechnology, the development of new generations of photosensitizers, and more advanced light delivery technologies are key elements in the future development of PDT as an effective cancer therapy. All clinical cases are summarized in Table 6.

## 5. Conclusions

PDT is a field of medicine that has undergone extraordinary development from its beginnings, based on simple observations of the impact of light on health, to advanced techniques for treating complex diseases, including cancer. PDT uses the unique properties of PSs, which, when exposed to the appropriate wavelength, emit ROS, leading to the selective destruction of pathological cells. Over the past decades, PDT has gained wide acceptance in the treatment of various diseases, such as skin cancer, head and neck cancer, bladder cancer, and gastrointestinal cancer.

One of the key achievements in PDT was the introduction of new generations of PSs, which are characterized by a greater specificity towards cancer cells and a shorter period of photosensitization, which significantly increases patient comfort and reduces side effects. In addition, the development of laser technologies and imaging techniques has enabled the precise delivery of light energy to target tissues, minimizing the damage to healthy cells and increasing the therapeutic efficacy.

Despite the significant progress, PDT still faces numerous challenges that require further optimization. The limited ability of light to penetrate deep into tissues remains one of the main limitations, especially in the treatment of deep-seated tumors. It is necessary to develop new methods of light delivery, such as the use of longer wavelengths, fiber optic technologies, or light-enhancing agents, to effectively treat deeper lesions. Long-term skin photosensitization after the use of some PSs is a significant clinical problem, limiting the quality of life of patients after therapy. The use of new generations of PSs with a shorter activity period and lower toxicity could significantly improve the safety and acceptability of PDT.

In addition, there is a need to develop more selective and effective PSs that would precisely target cancer cells with minimal impact on healthy tissues. Research on photosensitizers with a higher specificity that can be activated only in pathological cells could significantly increase the effectiveness of therapy. Combining PDT with other treatment modalities, such as immunotherapy, gene therapies, or targeted therapies, can lead to synergistic effects, improving treatment outcomes and reducing the risk of disease relapse.

There is still a lack of uniform, standardized PDT treatment protocols for different types of cancers and diseases. The introduction of standard guidelines for dosage, choice of photosensitizers, wavelength, and exposure time could contribute to improving clinical outcomes and facilitate the implementation of PDT in everyday medical practice. Further research into the molecular and cellular pathways of the response to PDT may open up new possibilities for optimizing this therapy. Understanding these processes at a deeper level will allow for the better adaptation of therapy to individual patient needs and the specificity of their diseases.

To sum up, PDT is a dynamically developing field, the therapeutic potential of which is constantly increasing as a result of new scientific and technological discoveries. PDT is a promising therapeutic option for many conditions, offering the benefits of minimal invasiveness, reusability, and the precise targeting of diseased tissues. The future of PDT depends on innovations in the light penetration, selectivity, and efficacy of PSs, and the integration with other treatment modalities. Focusing on these aspects can significantly improve the efficacy and safety of PDT, opening up new therapeutic possibilities in medicine.

## Figures and Tables

**Table 3:** Properties of some photosensitizing dyes approved for the treatment of PDT and used in PDT-related clinical trials.

Compound	Name	Absorption [nm]	Application	General Reviews
Porfimer sodium salt	Photofrin^®^	632	Canada (1993)—bladder cancer USA (1995)—esophageal cancer USA (1998)—lung cancer USA (2003)—Barrett’s esophagus Japan—cervical cancer Europe, Canada, Japan, USA, Great Britain—endobronchial cancer	-
5-aminolevulinic acid (ALA)	Levulan^®^	632	USA (1999)—actinic keratosis	[120]
Methyl aminolevulinate (MAL)	Metvix^®^	-	USA (2004)—actinic keratosis	[121,122]
Hexaminolevulinate (HAL)	Cysviev^®^	-	USA (2010)—diagnosis of bladder cancer	[123,124]
A derivative of benzoporphyrin Monoacid ring A (BPD-MA)	Visudyne^®^	689	USA (1999)—age-related macular degeneration	[125]
Meta-tetra(hydroxyphenyl)chlorin (m-THPC)	Foscan^®^	652	Europe—neck and head cancer	[126]
Ethyl ethiopurpurin	Purlytin^®^	664	Clinical trials—breast adenocarcinoma, basal cell carcinoma, Kaposi’s sarcoma, age-related macular degeneration	-
N-aspartyl chlorin e6 (NPe6)	Laserphyrin, Litx^®^	664	Japan (2003)—lung cancer	[127]
2-(1-hexyloxyethyl)-2-devinyl pyropheophorbide- (HPPH)	Photochlor^®^	665	Clinical trials—esophageal cancer, basal cell carcinoma, lung cancer, Barrett’s esophagus	[128]
Palladium-bacteriopheophorbide (WST09)	Tookad^®^	763	Clinical trials—prostate cancer	[129,130]
WST11	Stakel^®^	-	Clinical trials—prostate cancer	[130,131]
Motexafin luetium (Lu-Tex)	Lutrin, Optrin, Antrin^®^	732	Clinical trials—prostate cancer, age-related macular degeneration, breast cancer, cervical cancer, arterial disease	-
Tetrasulfonic aluminium phthalocyanine (APkS4)	Photosens^®^	676	Russia (2001)—stomach, skin, lips, oral cavity, tongue, breast cancer	[132]
Silicon phthalocyanine (Pc4)	-	675	Clinical tests—practical keratosis, Bowen’s disease, skin cancer, mycosis	[133]

**Table 4:** The most important centers specializing in skin PDT in the world. All on lie addresses were accessed on 13 September 2024.

Country	Research Centre	Internet Adress
Austria	Wiener Privatklinik Rudolfinerhaus Privatklinik Döbling Private Clinic Akh Vienna General Hospital Graz Ragnitz Private Clinic Private Clinic Konfraternetat Leech Pivate Clinic	https://wiener-privatklinik.com/ https://www.rudolfinerhaus.at/ https://www.privatklinik-doebling.at/ https://www.akhwien.at/ https://www.privatklinik-graz-ragnitz.at/ https://www.privatklinik-confraternitaet.at https://sanlas.at/einrichtungen/privatklinik-leech/
China	Fuda Cancer Hospital	https://www.fudahospital.com/
Czech Republic	Motol University Hospital University Hospital Brno	https://www.fnmotol.cz/ https://www.fnbrno.cz/
France	American Hospital of Paris Oncological Institut Gustave Roussy	https://www.american-hospital.org/ https://www.gustaveroussy.fr/
Germany	Dermatologikum Berlin Solingen City Hospital University Hospital Rechts der Isar Nuremberg Hospital Helios Medical Group University Hospital of Köln Charité University Hospital Frankfurt University Hospital Essen University Hospital Meoclinic	https://dermatologikum-berlin.de/ https://www.klinikumsolingen.de https://www.mri.tum.de/ https://www.klinikum-nuernberg.de/ https://www.helios-international.com/en https://www.uk-koeln.de/ https://www.charite.de/ https://www.unimedizin-ffm.de/ https://www.uk-essen.de/ https://www.meoclinic.de/en/
India	Gleneagles Global Hospitals BLK Super Speciality Hospital Apollo Hospitals Fortis Hospital Manipal Hospitals Group Medanta Hospital Wockhardt Hospitals	https://www.gleneagleshospitals.co.in/ https://www.blkmaxhospital.com/ https://www.apollohospitals.com/ https://www.fortishealthcare.com/ https://www.manipalhospitals.com/ https://www.medanta.org/ https://international.wockhardthospitals.com
Israel	Sourasky Medical Center (Ichilov) Assuta Medical Center Sheba Medical Center Rambam Hospital Herzliya Medical Center Hadassah Medical Center Yitzhak Rabin Medical Center Schneider Children’s Medical Center Shaare Zedek Medical Center Ramat Aviv Medical Center Assaf Harofeh Medical Center Meir Medical Center	https://www.tasmc.org.il/ https://www.assuta.co.il/ https://www.shebaonline.org https://www.rambamhcc.com/ https://hmcisrael.com/ https://hadassahmedical.com/ https://rabin-medical.org/ https://www.schneider.org.il/ https://www.szmc.org.il/ https://en.mcra.co.il/ https://www.shamir.org/ http://www.mmc.org.il/
Italy	Salvator Mundi International Hospital	https://upmc.it/
Korea	JK Plastic Surgery Clinic Asan Medical Center Severance Hospital Gachon University Gil Medical Center Cheil General Hospital & Women’s Healthcare Center Kyung Hee University Hospital (KUIMS) Seoul National University Hospital Soon Chun Hyang University Hospital Inha University Hospital Chung-Ang University Hospital	https://www.jkplastic.com/ https://www.amc.seoul.kr/ https://sev.severance.healthcare/sev/index.do https://www.gilhospital.com/web/www/home http://www.gmcheil.co.kr/en.html https://www.khmc.or.kr/en/main.do https://www.snuh.org/intro.do https://international.schmc.ac.kr/ https://www.inha.com/page/main https://ch.cauhs.or.kr/
Lithuania	Abromiskes Rehabilitation Center Medical Diagnostic and Treatment Centre	https://www.abromiskes.lt/ https://www.hila.lt/en/
Poland	Krakow University Hospital	https://www.su.krakow.pl/
Singapore	Raffles hospital	https://www.rafflesmedicalgroup.com/services/hospital/
Spain	Hospital Quirónsalud Barcelona Clínica Universidad de Navarra	https://www.quironsalud.com/hospital-barcelona https://www.cun.es/
Thailand	Bumrungrad International Hospital	https://www.bumrungrad.com/en
Turkey	Medicana Hospitals Group Medistate Hospital Medipol Mega University Hospital Memorial Hospital Medical Park Hospitals Group Koc University Hospital Acibadem Maslak Hospital Liv Hospital Anadolu Medical Center Hisar Intercontinental Hospital Gaziosmanpasa Private Clinic Memorial Şişli Hospital Medical Park Gebze Clinic Memorial Bahçelievler Hospital Memorial Hospital Ataşehir Medical Park Göztepe Hospital Acibadem Taksim Clinic Medical Park Fatih Hospital	https://www.medicanainternational.com/ https://www.medistate.com.tr/en/home https://medipol.com.tr/ https://www.memorial.com.tr/ https://medicalparkinternational.com/ https://www.kuh.ku.edu.tr/en https://www.acibadem.com.tr/ https://www.livhospital.com/en https://www.anadolumedicalcenter.com/en https://hisarhospital.com/en/ https://www.gophastanesi.com.tr/ https://www.memorial.com.tr/ https://www.medicalpark.com.tr/ https://www.memorial.com.tr/ https://www.memorial.com.tr/ https://www.medicalpark.com.tr/ https://www.acibadem.com.tr/ https://medicalparkinternational.com/

**Table 5:** Summary of clinical progress.

Year/Years	Major Clinical Achievements
1900s	Discovery of photodynamic therapy (PDT) using light to activate chemical substances to kill microorganisms and malignant cells, laying the foundation for modern PDT.
1960s	Therapeutic use of hematoporphyrin derivatives (HPDs), which selectively accumulate in tumors, establishing PDT’s role in cancer treatment.
1970s	The first clinical applications of PDT using HPD, particularly in the treatment of skin cancer, began.
1993	Photofrin^®^ approved in Canada for bladder cancer treatment, leading to international expansion and approvals in the Netherlands, France, Germany, and Japan for various cancers.
1995	FDA approval of Photofrin^®^ for treating esophageal cancer, following clinical trials that demonstrated its effectiveness compared to Nd-YAG laser treatment.
1999	FDA approval of verteporfin (Visudyne^®^) for treating age-related macular degeneration (AMD), stabilizing choroidal neovascularization.
2001	Foscan^®^ (temoporfin) approved by the EMA for advanced squamous cell carcinoma of the head and neck, although the FDA did not approve it.
2003	PDT using verteporfin to treat angiocellular hemangioma showed promising results, with significant vision improvement and no recurrences or side effects.
2006	Studies showed that PDT with Photofrin^®^ increases vascular endothelial growth factor (VEGF) expression in lung cancer, enhancing its therapeutic effects when combined with inhibitors.
2009	PDT effectively treated nasopharyngeal cancer (NPC), demonstrating effective tumor growth inhibition and minimal side effects.
2011	A pioneering study used PDT to treat potentially malignant oral diseases, achieving complete response in 81% of patients with conditions like leukoplakia and erythroplakia.
2015	PDT with MAL and imiquimod cream (IMIQ) showed equal effectiveness in preventing new non-melanoma skin cancers (NMSCs), with patients preferring PDT for ease of use.
2016	PDT using HPPH for early-stage laryngeal diseases demonstrated good response rates and established the maximum tolerated dose (MTD) in clinical settings.
2017	PDT showed significant improvements in patients with vulvar lichen sclerosus, achieving 87.25% improvement, especially in reducing erosion and hyperkeratosis.
2018	ALA-PDT found to be an effective and safe treatment for rosacea, providing long-term relief and eliminating symptoms.
2019	PDT combined with vertebroplasty or kyphoplasty was used to treat vertebral metastases, showing technical feasibility and pain reduction in patients.
2020	Modified PDT for genital warts proved nearly painless while maintaining high effectiveness, a breakthrough in pain management for PDT.
2021	A study compared PDT and trichloroacetic acid (TAA) for treating HPV warts around the anus and vulva, showing lower recurrence rates with PDT.
2022	PDT with 5-ALA was shown to significantly reduce clinical symptoms of nicotine stomatitis in smokers, demonstrating its effectiveness as a non-invasive treatment.
2023	PDT using indocyanine green (ICG-PDT) effectively inhibited keloid fibroblast activity and induced autophagy and apoptosis, suggesting potential for treating keloids.
2023	Intranasal PDT was found to reduce SARS-CoV-2 infectivity in mildly symptomatic patients, contributing to pandemic treatment strategies.

**Table 6:** Summary of the clinical cases.

References	Year	Brief Description	Outcome
[27]	1993	Canada approves Photofrin^®^ for the prophylactic treatment of bladder cancer, marking the start of its international expansion. Also approved in the Netherlands, France, Germany, and Japan for various cancers, including lung, esophageal, and cervical cancer.	International expansion and broader use of the drug.
[27]	1993	Canada approves Photofrin^®^-PDT after surgical removal of bladder tumors for patients at high risk of recurrence. Preliminary study results were presented in 1991.	Reduced risk of cancer recurrence.
[28]	1994	In a study of 34 patients, disease recurrence was 81% without PDT and 39% with PDT. Average time to recurrence: 91 days (control) and 394 days (PDT). One-third experienced photosensitivity, and 93% had urinary symptoms. Lower doses suggested to reduce side effects.	Reduced recurrence, but significant side effects led to suggestions for therapy adjustments.
[29]	1995	Photofrin^®^ received FDA approval after phase three clinical trials in the US. A multi-center study compared PDT and Nd-YAG laser ablation for obstructive esophageal cancer. Both reduced dysphagia similarly, but PDT had longer tumor response and more complete responses. PDT had fewer procedures, but more adverse reactions. Fewer perforations occurred with PDT (1% vs. 7% for Nd-YAG).	Both treatments were equally effective. PDT was easier to perform, caused fewer perforations, but had more adverse reactions.
[30]	1996	Biel published a study on early-stage head and neck cancer treatment with Photofrin^®^, covering various cancer types. Complete response was achieved in all 22 patients with superficial laryngeal cancer. Similar results were found in oral and nasopharyngeal cancer. Some recurrences were observed in laryngeal/tracheal papilloma patients.	High success rate, but recurrences in papilloma patients and mild-to-severe pain, controllable with oral analgesics.
[31]	1997	Largest PDT study using Photofrin^®^ on 55 patients with superficial esophageal cancer, often linked to Barrett’s esophagus. After six months, 24 of 36 patients with high-grade dysplasia showed no dysplasia, and 7 had no residual Barrett’s esophagus. Complications included esophageal stricture (29 patients), but PDT had lower mortality (0%) than surgery (6–14%).	PDT significantly reduced dysplasia with fewer risks and costs than surgery, although stricture complications were common.
[32]	1999	A new era in the treatment of age-related macular degeneration (AMD) began with FDA approval of PDT with verteporfin (Visudyne^®^) for patients with predominantly classic subfoveal neovascularization. Phase I–II trials showed it could stabilize CNV leakage for up to 3 months, with successful long-term results in phase III trials.	FDA approval for AMD treatment with verteporfin, successfully stabilizing vision loss for thousands of patients.
[33,34,35]	1999	PDT made significant advancements in dermatology, particularly for treating solar keratoses, common skin lesions. Traditional methods like cryosurgery and laser ablation were replaced by 5-ALA-PDT and MAL-PDT, achieving high effectiveness (89–92%) in eliminating lesions, especially on the face and scalp.	High efficacy in removing solar keratoses with PDT, especially on the face and scalp.
[36]	2001	Foscan^®^, a drug used in PDT, was approved by the EMA for treating advanced squamous cell carcinoma of the head and neck. It was submitted to the FDA in 2000 but was not approved. Foscan^®^ has been studied for various cancers, but its high potency can cause damage to healthy tissue and carries the risk of skin burns from photosensitizer extravasation.	Approved in Europe for head and neck cancer; potent but with notable risks of tissue damage and skin burns.
[37]	2003	A prospective, non-randomized study on PDT using verteporfin for 19 patients with symptomatic angiocellular hemangioma. Treatment sessions ranged from 1 to 5, and the average follow-up time was 10.6 months. The study showed promising results, with vision improving in 73.3% of patients and complete resolution of exudation in 94.8% of cases. No recurrences or adverse effects were reported.	Promising outcomes in treating angiocellular hemangioma with verteporfin-PDT, with vision improvement and no recurrences or adverse effects.
[38]	2004	Clinical trials reported using PDT for cholangiocarcinoma (CC), with nearly two-thirds of patients dying from progressive CC and associated complications. Despite high mortality rates, repeated PDT treatments for segmental biliary obstructions showed a significant increase in median survival, ranging from >9 to 16.2 months. Even patients in poor condition benefited from the treatment.	Significant increase in survival for cholangiocarcinoma patients treated with PDT, despite high mortality from advanced disease and complications.
[39]	2005	Scientists from the Weizmann Institute in Israel developed a water-soluble derivative of Tookad^®^, named WST-11 (later Stakel^®^ and Padeliporfin). This compound caused rapid vascular shutdown during photodynamic therapy (VTP) via a type I photochemical process. WST-11 was produced by Steba Biotech and marked a key step in PDT development.	Breakthrough in PDT with WST-11, enabling more effective vascular-targeted photodynamic therapy (VTP).
[40]	2006	PDT using Photofrin^®^ was shown to increase the expression of VEGF and prostaglandin E2 in murine tumors. Combining PDT with VEGF or cyclooxygenase-2 inhibitors increased therapeutic effectiveness. Inhibiting matrix metalloproteinases (MMPs) further enhanced the antitumor effects of PDT in vivo.	Enhanced effectiveness of PDT in lung cancer treatment through combination therapies with VEGF and MMP inhibitors.
[41]	2007	PDT began to gain importance in microbiology. Smijs’ research team used an ex vivo human skin model to test porphyrins’ ability to eliminate *T. rubrum*, a dermatophyte. Short incubation periods (8 h) led to complete fungus destruction post-irradiation, but longer incubation (>24 h) did not.	Effective elimination of *T. rubrum* with PDT and porphyrins under short incubation periods.
[42]	2007	A study treated 15 patients with histologically confirmed actinic cheilitis using PDT with MAL. After two treatment sessions, complete clinical remission was observed in almost half the patients, but histopathological examination showed signs of dysplasia in most, possibly due to uneven absorption of the photosensitizing agent.	Partial clinical remission observed, but uneven agent absorption led to continued signs of dysplasia in many cases.
[43]	2006	A European, randomized, multicenter, placebo-controlled trial compared PDT with MAL, cryotherapy, and 5-FU in patients with Bowen’s disease. PDT with MAL achieved the highest rate of complete remission at 12 months.	PDT with MAL showed the best results in treating Bowen’s disease compared to other methods.
[44]	2007	Zane’s research showed that PDT can affect collagen fibers, suggesting the possibility of stimulating collagen synthesis. PDT also led to the reorganization or accumulation of new collagen fibers, potentially improving skin texture.	PDT may stimulate collagen synthesis and improve skin texture by reorganizing collagen fibers.
[45]	2009	PDT was recognized as a potentially effective treatment for nasopharyngeal cancer (NPC) without the severe side effects of radiotherapy. Studies showed that first-generation PS (hematoporphyrin) was effective, but second-generation PS (temoporfin) was more effective. A new light delivery applicator was developed to address the challenges of nasopharyngeal illumination.	PDT effectively treated NPC, with fewer side effects than radiotherapy. Temoporfin was shown to be more effective than hematoporphyrin.
[46]	2010	A study on the use of PDT for anal cancer was conducted. The procedure was well-tolerated, performed on an outpatient basis, and had no major complications. Patients experienced manageable pain, and, by the end of the first month, no patient required pain medication. All patients showed no evidence of disease (NED) at 3–4 months, and no local failure or sphincter damage was observed at the 18–48-month follow-up.	PDT effectively treated anal cancer with no major complications, and all patients showed no evidence of disease.
[47]	2011	A prospective study on 147 patients with potentially malignant oral diseases treated with 5-ALA or mTHPC-PDT. The follow-up (7.3 years) compared recurrence and malignant transformation rates. Complete response was observed in 119/147 patients (81%). Malignant transformation occurred in 7.5% of patients, mainly in cases of erythroplakia and heterogeneous leukoplakia.	5-ALA-PDT and mTHPC-PDT showed high effectiveness, with 81% complete response and a low malignant transformation rate.
[48]	2011	A study involving 15 patients with treatment-resistant acuminal papilloma treated with ALA-PDT. Complete recovery was seen in 9 of 15 patients after five PDT sessions. The study showed rapid remission of lesions in the anal area and activation of specific immunity (CD4+ T cells and dendritic cells) in the affected skin.	ALA-PDT showed effectiveness in treating resistant acuminal papilloma, particularly in the anal area, with immune cell activation playing a role in recovery.
[49]	2013	A randomized, comparative study on the treatment of peri-implantitis. The study involved 20 patients and 20 controls to compare the antibacterial effectiveness of PDT with surgical therapy for peri-implantitis in patients with dental implants.	PDT showed significant reduction in bleeding and inflammatory secretions compared to surgical therapy, but no significant difference in total anaerobic bacteria.
[50]	2015	A study comparing the effectiveness and safety of MAL-PDT with imiquimod cream (IMIQ) 5% in preventing new non-melanoma skin cancers (NMSCs), including actinic keratoses (AK), in patients with field lesions on the face or scalp.	Both treatments were safe and effective in preventing new AKs, with patients preferring MAL-PDT due to response rates and ease of the procedure.
[51]	2016	An open-label, non-comparative study evaluating the safety of PDT using 3-(1′-hexyloxyethyl) pyropheophorbide-a (HPPH) in treating early-stage laryngeal diseases, including dysplasia, carcinoma in situ, and T1 squamous cell carcinoma (SCC).	HPPH-PDT therapy was generally safe and effective, with an 82% response rate in T1 SCC patients. Transient hoarseness was common, while severe edema requiring tracheostomy occurred in two cases.
[52]	2016	A study conducted in eight hospitals in China to investigate the effectiveness and safety of PDT using hemoporfin and a 532 nm laser in the treatment of port-wine stain. The study included patients aged 14 to 65 and assessed improvements at weeks 8 and 16.	PDT-hemoporfin showed significantly better results compared to placebo, with nearly 90% of patients achieving at least some improvement. Hyperpigmentation was reported in ~23% of patients.
[53]	2017	A study on the effectiveness of PDT in treating vulvar lichen sclerosus, a chronic and incurable disease. In total, 102 patients aged 19 to 85 received 5-ALA PDT treatments once a week for 10 weeks, with irradiations using a PhotoDyn 501 halogen lamp.	PDT showed an 87.25% improvement rate, particularly effective in reducing petechiae, telangiectasia, erosion, and cracks; but less effective in reducing atrophic changes. Good cosmetic outcomes were also observed.
[54]	2018	A study on the effectiveness and safety of ALA-PDT in treating erythematotelangiectatic and papulopustular rosacea in Chinese patients with Fitzpatrick skin types III and IV. Treatments were repeated every 10 days for 10 weeks.	All patients showed gradual improvement, with complete resolution of clinical symptoms by 24 weeks. Side effects were transient and tolerable. ALA-PDT was found to be an effective and safe method for treating rosacea.
[55]	2019	A pioneering study investigating the use of PDT as a tumor ablation method combined with vertebroplasty (VP) and balloon kyphoplasty (KP) for vertebral compression fractures (VCF) caused by vertebral metastases. The study evaluated safety and clinical outcomes in 30 patients.	Vertebral PDT as an adjunct to VCA was found to be safe and technically feasible. Significant pain reduction was observed in the 50 and 100 J/cm groups. No complications were directly linked to PDT.
[56]	2019	A study assessing clinical and microbiological periodontal parameters after antibacterial PDT (APDT) and scaling and root planning (SRP) in HIV-infected and -uninfected patients with necrotizing ulcerative periodontitis (NUP).	APDT improved periodontal parameters and reduced bacterial levels in both HIV-infected and -uninfected patients. APDT showed greater PD reduction and CAL gain, with decreased levels of Aa, Tf, and Pg.
[57]	2020	A prospective, randomized study comparing modified photodynamic therapy (M-PDT) and coherent photodynamic therapy (C-PDT) in treating genital warts, focusing on effectiveness, pain, and safety. Twenty patients completed the study.	M-PDT and C-PDT showed similar cure and recurrence rates. However, M-PDT was significantly less painful, marking a breakthrough in pain management during PDT treatments.
[58]	2021	A study comparing methylene blue (MB) photodynamic therapy with intense pulsed light (IPL) for the treatment of warts. Patients were divided into three groups: MB/IPL/PDT therapy, IPL-only, and a control group.	MB/IPL/PDT therapy showed a cure rate of 40.9%, significantly higher than IPL-only (23.4%). ImageJ analysis confirmed greater wart reduction in the MB/IPL/PDT group.
[59]	2021	A randomized, controlled clinical trial comparing PDT with trichloroacetic acid (TAA) for the treatment of HPV warts around the anus and vulva; 16 patients received PDT and 15 received TAA.	PDT had a 63% cure rate and 0% recurrence, while TAA had a 60% cure rate and 33% recurrence. PDT demonstrated potential immune modulation and reduced viral load, leading to lower recurrence.
[60]	2022	A study evaluating antimicrobial PDT (aPDT) as an adjunct to topical antiviral therapy (TAT) in children with herpetic gingivostomatitis. The study involved 45 children, divided into three groups: TAT, aPDT, and TAT + aPDT.	All groups showed reduced pain, HSV-1 load, and cytokine levels, with TAT + aPDT showing statistically significant improvement over TAT or aPDT alone.
[61]	2022	A study evaluating the use of 5-ALA-PDT in treating nicotine stomatitis in smokers; 24 patients were divided into a test group (5-ALA-PDT) and a control group (smoking cessation). Treatment and follow-up were performed over 8 weeks.	5-ALA-PDT significantly reduced clinical symptoms of nicotine stomatitis, showing greater improvement than smoking cessation alone. No negative side effects were reported.
[62]	2023	A study comparing nasal decolonization methods in hemodialysis patients carrying Staphylococcus aureus, using PDT with methylene blue vs. mupirocin treatment. Both methods were effective in eliminating *S. aureus* immediately after treatment.	Both methods were effective, but 67% of patients in the PDT group were recolonized within 3 months, while no adverse effects were reported. Larger studies are needed to compare long-term efficacy.
[63]	2023	A study investigating the therapeutic potential of PDT with indocyanine green (ICG-PDT) in treating keloids, focusing on the inhibition of cellular activity and migration of keloid fibroblasts.	ICG-PDT inhibited keloid fibroblast activity, induced autophagy and apoptosis, and reduced collagen synthesis, showing promise for keloid treatment at low drug concentrations.
[64]	2023	A randomized, placebo-controlled clinical trial evaluating the effectiveness of intranasal PDT in shortening the infectious period of SARS-CoV-2 carriers with mild symptoms, and its impact on immune response.	Intranasal PDT was found to be safe, reduced SARS-CoV-2 infectivity, and slowed the decline of specific immune responses in mildly symptomatic COVID-19 patients.
